# An AIE-active polyvinyl alcohol/berberine/hemoadhican smart hydrogel for scarless wound healing with visual pH monitoring

**DOI:** 10.1016/j.mtbio.2026.103224

**Published:** 2026-05-10

**Authors:** Rui Fang, Simeng Chen, Qiaozhen Liu, Mingxiong Zhang, Xi Xu, Jianfa Zhang

**Affiliations:** a Center for Molecular Metabolism, Nanjing University of Science & Technology, 200 Xiaolingwei Street, Nanjing, 210094, China; b Key Laboratory of Metabolic Engineering and Biosynthesis Technology, Ministry of Industry and Information Technology, China

**Keywords:** Hemoadhican, Scarless wound healing, Hydrogel, pH monitoring

## Abstract

Hard-to-heal wounds characterized by infection, dysregulated inflammation, and fibrosis remain a significant clinical challenge. This is particularly true for chronic wounds, such as those resulting from burn injuries and diabetic ulcers. Here, a physical self-assembly strategy is employed to co-incorporate berberine and the polysaccharide hemoadhican into a polyvinyl alcohol matrix, generating a multifunctional hydrogel that integrates antibacterial activity, immunomodulation, and real-time wound pH monitoring. The hydrogel exhibits robust mechanical stability, enabling adaptation to dynamic wound microenvironments. *In vitro* studies demonstrate that the hydrogel promotes fibroblast migration and angiogenesis, effectively scavenges reactive oxygen species, and regulates macrophage polarization toward an anti-inflammatory M2 phenotype. *In vivo*, the hydrogel accelerates wound closure in rabbit ear scar models, diabetic infected wounds, burn wounds, and mixed bacterial infection models, while reducing bacterial burden, enhancing skin appendage regeneration, and attenuating fibrosis and scar formation. Notably, aggregation-induced emission responsiveness allows visual monitoring of wound pH changes, providing intuitive feedback on wound status during healing. Together, this work demonstrates an integrated diagnostic–therapeutic hydrogel platform and offers a material design paradigm for scar-free wound repair.

## Graphical abstract

**Figure:**
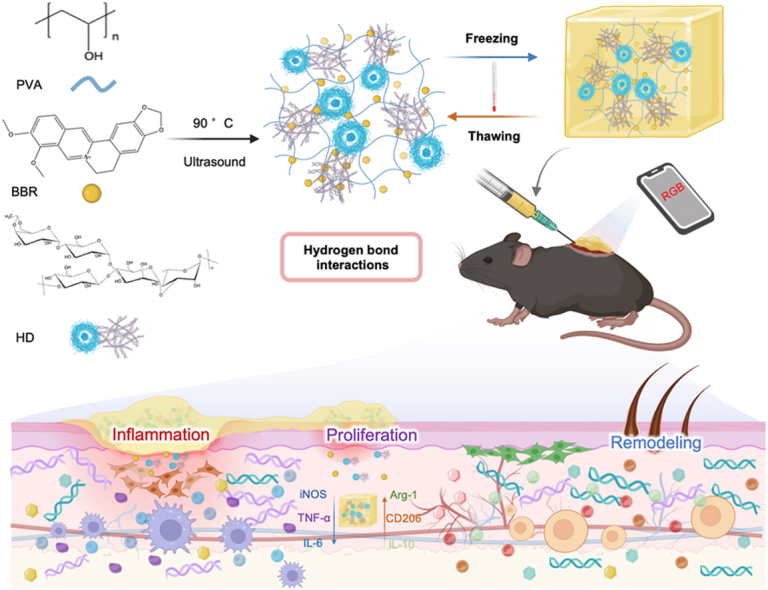

## 1 Introduction

Skin wound repair is a highly dynamic process orchestrated through the coordinated regulation of immune responses, cell migration and proliferation, angiogenesis, and extracellular matrix (ECM) remodeling [1,2]. However, under conditions of severe skin injury, this finely tuned regulatory process is often disrupted, resulting in abnormal ECM deposition, excessive fibroblast activation, and increased collagen production, which collectively contribute to pathological scar formation. In recent years, various functional materials and therapeutic strategies have been developed to promote wound repair. For example, nano-diamond-enhanced zinc oxide (ND-ZnO) enhances wound healing by promoting dermal fibroblast migration and collagen synthesis [3]. Similarly, 3D-printed alginate/graphene scaffolds regulate reactive oxygen species levels to promote cell migration and angiogenesis, demonstrating promising applications in tissue repair [4]. Furthermore, photobiomodulation stimulates adipose tissue with light, enhancing collagen production and improving healing quality [5]. Nitric oxide (NO), a small gaseous signaling molecule characterized by rapid diffusion and metabolic activity, contributes to wound repair through multiple mechanisms: promoting local blood flow, inducing angiogenesis, enhancing collagen deposition, accelerating re-epithelialization, and exerting anti-inflammatory, antioxidant, and broad-spectrum antibacterial effects [[6], [7], [8]]. However, persistent inflammatory responses triggered by bacterial infections or other pathological factors can significantly prolong the healing process and promote the formation of fibrotic scars [9,10]. Clinically, such scars are typically characterized by rigid elevations, compromised mechanical properties, and pigmentary abnormalities, frequently accompanied by pruritus, chronic pain, and restricted mobility. Beyond physical impairment, scarring can also induce psychological distress, including anxiety and depression, severely affecting the quality of life of nearly 100 million patients worldwide [[11], [12], [13], [14]]. Current therapeutic strategies for scar management—including silicone gel application, corticosteroid injections, laser ablation, radiotherapy, and surgical excision—are limited by suboptimal efficacy and high recurrence rates, highlighting an unmet clinical need [15,16]. Increasing evidence suggests that early intervention during wound healing, aimed at modulating inflammation, fibrosis, and tissue remodeling, is one of the most effective approaches to preventing pathological scarring [17,18]. Therefore, developing a therapeutic system capable of simultaneously addressing infection control, immune homeostasis, and regenerative repair throughout the entire wound-healing process is a critical step toward achieving scar-free skin regeneration.

The pH of the wound microenvironment is a critical parameter in wound diagnosis and is closely associated with healing outcomes [[19], [20], [21], [22]]. Clinical evidence indicates that pH serves not only as a reliable biomarker for delineating wound-healing stages but also that wounds failing to shift toward an acidic milieu during the early healing phase exhibit a significantly higher risk of healing failure and subsequent skin grafting [23] Accordingly, real-time monitoring of pH dynamics during wound repair facilitates the evaluation of healing progression and provides essential guidance for developing individualized therapeutic strategies. Berberine (BBR), an isoquinoline alkaloid derived from traditional Chinese medicinal plants, exhibits well-established broad-spectrum antibacterial activity and possesses pH-responsive aggregation-induced emission (AIE) characteristics. These properties make BBR a promising candidate for wound dressings that combine antibacterial functionality with real-time monitoring capabilities [24,25]. Hemoadhican (HD) is an extracellular polysaccharide produced by *Paenibacillus* sp. 1229, characterized by a hexosyl repeating unit structure: →)-α-L-Rhap-(1 → 3)-β-D-Glcp-(1 → 4) [4,6-ethylidene-α-D-Galp-(1 → 4)-α-D-Glcp-(1 → 3)]-α-D-Manp-(1→ [26]. Previous studies have demonstrated that HD-based Janus patches exhibit potent anti-inflammatory activity by effectively suppressing the overexpression of pro-inflammatory mediators, reducing aberrant collagen deposition, and improving the local inflammatory microenvironment, thereby showing potential for promoting scar-free healing [27]. Based on these properties, we hypothesize that the stable integration of BBR and HD into a unified delivery system could simultaneously enable sustained antibacterial activity, pH-responsive monitoring, suppression of excessive inflammation, and inhibition of fibrotic remodeling within complex wound microenvironments, ultimately promoting functional skin regeneration with near-native tissue architecture. However, HD lacks chemically reactive functional groups, making efficient and controllable covalent conjugation with BBR highly challenging while preserving biological activity [26,28]. Additionally, BBR suffers from intrinsic pharmacokinetic limitations, including low bioavailability, poor aqueous solubility and stability, and rapid metabolism, which substantially restrict its clinical translation as an active wound dressing component [29]. Moreover, within the dynamic and moist wound environment, both BBR and HD are susceptible to rapid leaching from the wound surface, leading to insufficient retention and compromised long-term efficacy. Consequently, developing a functional biomaterial platform capable of efficiently loading, stably anchoring, and sustainably delivering BBR and HD represents a critical challenge for realizing their synergistic therapeutic potential.

Hydrogels are widely recognized as promising soft and moist biomaterials due to their high water content, tunable mechanical properties, and close resemblance to the natural ECM in both microstructural and biochemical characteristics. Consequently, they have been extensively investigated as drug delivery platforms for wound healing applications [[30], [31], [32]]. Their three-dimensional polymeric networks provide an ECM-mimetic microenvironment that supports cell migration and proliferation, while also enabling the controlled release of antimicrobial agents, anti-inflammatory factors, and other bioactive molecules. This makes hydrogels critical carriers for precision therapy in chronic wound management [33,34]. However, conventional hydrogel dressings still face significant limitations, including slow or uncontrollable degradation, limited functionality, lack of real-time monitoring capabilities, and insufficient adaptability to irregular wound geometries [[35], [36], [37], [38]]. Furthermore, some multifunctional hydrogel systems depend on complex bioactive components—such as chemokines, antimicrobial peptides, or exosomes—which present considerable challenges related to handling, storage stability, manufacturing scalability, and immunological safety, thereby hindering clinical translation [[39], [40], [41]]. Therefore, there remains an urgent need to develop multifunctional hydrogel dressings that are easy to use, cost-effective, and suitable for clinical implementation.

**Scheme 1:**
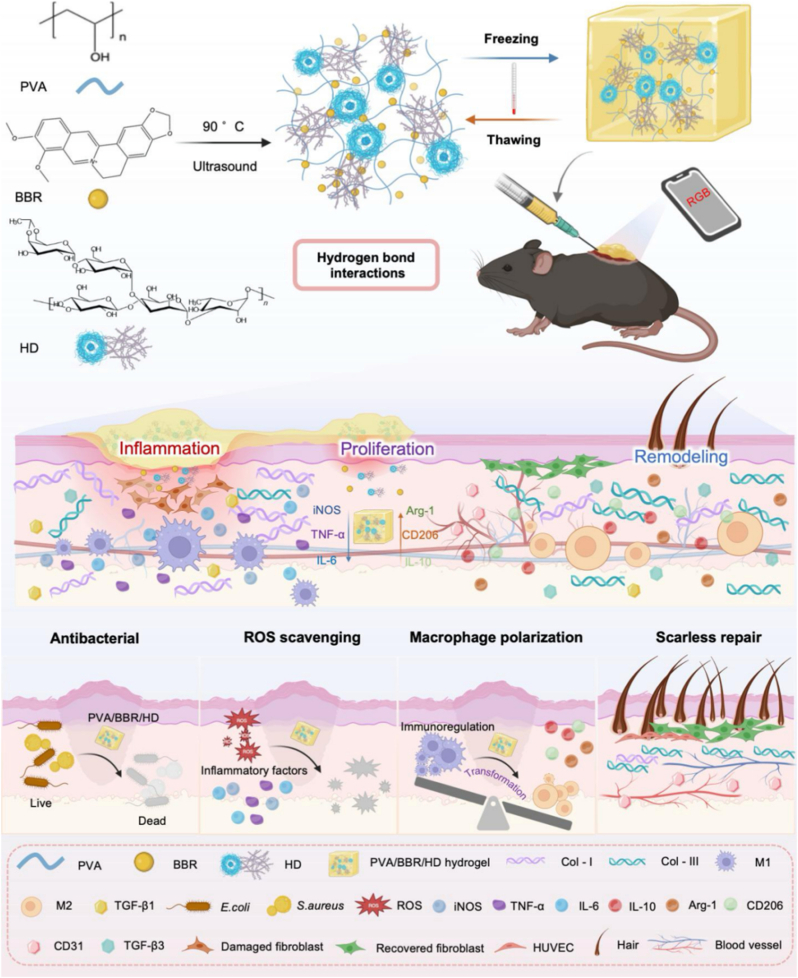
Schematic illustration of the fabrication of an injectable polyvinyl alcohol/berberine/hemoadhican (PVA/BBR/HD) hydrogel and its wound healing mechanism.

To overcome the translational challenges associated with the synergistic application of BBR and HD, polyvinyl alcohol (PVA) was selected as the hydrogel scaffold in this study. Using a physical self-assembly strategy, BBR and HD were simultaneously incorporated to construct a multifunctional PVA/BBR/HD hydrogel endowed with aggregation-induced emission (AIE) responsiveness, dual antibacterial and anti-inflammatory activities, and real-time wound pH monitoring capability. The therapeutic performance of the PVA/BBR/HD hydrogel was systematically evaluated across multiple representative wound models, including mixed bacterial infection wounds, deep burn injuries, chronic diabetic ulcers, and rabbit ear injury models. Compared with commercial dressings, this multifunctional hydrogel demonstrated significantly enhanced wound-healing efficacy *in vivo*. Specifically, it effectively reduced bacterial burden at the wound site, mitigated persistent inflammatory responses, and promoted angiogenesis and tissue regeneration, while simultaneously suppressing aberrant collagen deposition, attenuating fibrosis, and alleviating scar formation. Additionally, leveraging its intrinsic AIE-responsive properties, the hydrogel enables visual monitoring of wound pH dynamics, providing intuitive guidance for wound status assessment and optimization of treatment timing (Scheme 1). Collectively, the PVA/BBR/HD hydrogel offers an integrated strategy to address infection, inflammatory dysregulation, and fibrotic progression in diverse difficult-to-heal wounds, while also providing valuable insights into the design of smart multifunctional biomaterials with combined diagnostic and therapeutic functions. This work thus holds important implications for the future development of scar-free wound-healing materials.

## 2 Results and discussion

### 2.1 Preparation and characterization of PVA/BBR/HD hydrogels

**Fig. 1:**
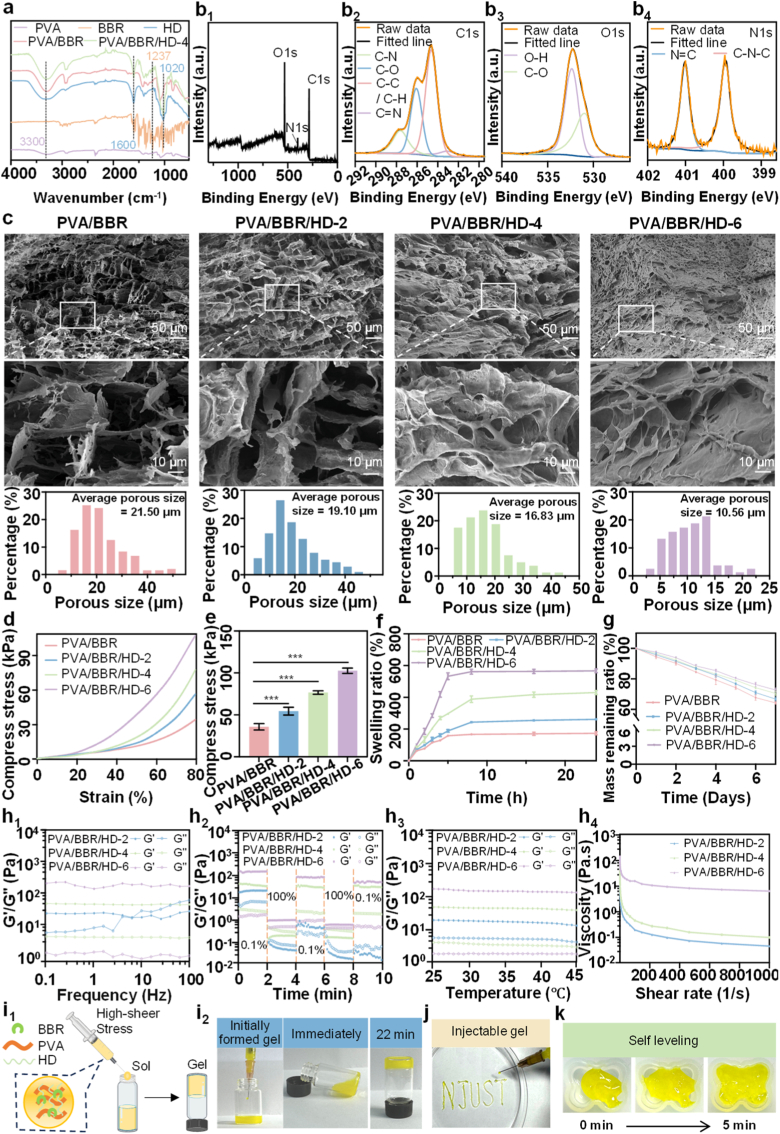
Characterization of PVA/BBR/HD hydrogels. (a) FTIR spectra. (b_1_) XPS full survey spectra, and high-resolution XPS spectra of (b_2_) C 1s, (b_3_) O 1s, and (b_4_) N 1s for the PVA/BBR/HD-4 hydrogel. (c) SEM images of PVA/BBR/HD hydrogels prepared with varying HD contents. (d) Compressive stress–strain curves of PVA/BBR/HD hydrogels. (e) Compressive stress at 80% strain (n = 3). (f) Swelling ratio and (g) degradation profiles of PVA/BBR/HD hydrogels. (h_1_) Storage modulus (G′) and loss modulus (G″) as functions of frequency (0.1–100 Hz). (h_2_) Cyclic strain sweep rheological test. (h_3_) Temperature-dependent storage and loss moduli (25–45 °C). (h_4_) Viscosity as a function of shear rate (0.01–1000 s^−1^) for PVA/BBR/HD hydrogels at different concentrations. (i_1_) Schematic illustration of injectability testing and (i_2_) photograph showing shear-thinning injectability. (j) Photograph demonstrating good printability and writability. (k) Self-leveling behavior of the PVA/BBR/HD-4 hydrogel. Statistical significance was analyzed using one-way ANOVA (*p*< 0.001, ∗). Error bars represent standard deviation.

During wound healing, early infection, mid-stage inflammatory imbalance, and late-stage fibrotic tendencies are critical factors that impede skin repair. To address these challenges, a PVA/BBR/HD self-assembled hydrogel dressing was developed via a freeze–thaw (F–T) cycling process for the integrated diagnosis and treatment of difficult-to-heal wounds. This design preserves the inherent bioactivity of HD while enhancing the bioavailability of BBR, thereby promoting wound repair through multifunctional synergistic effects. Fourier Transform Infrared (FTIR) spectra (Fig. 1a) display a characteristic peak at 3300 cm^−1^, corresponding to –OH stretching vibrations, confirming the presence of PVA. Peaks at 1237 cm^−1^, attributed to N–H and C–H bending vibrations of BBR, indicate its successful incorporation into the hydrogel [42]. In comparison, the PVA/BBR/HD-4 hydrogel exhibits additional absorption peaks at 1600 cm^−1^ and 1020 cm^−1^, corresponding to C=O stretching in HD and the polysaccharide heterocyclic carbon fingerprint region, respectively [28]. The intensities of these characteristic peaks increase with higher HD content (Fig. S1), suggesting uniform incorporation of HD into the PVA/BBR network. Enhanced –OH stretching vibrations in PVA/BBR/HD further indicate that HD's polyhydroxy structure strengthens hydrogen bonding interactions with water molecules, thereby improving the hydrogel network's integrity and stability [43]. X-ray photoelectron spectroscopy (XPS) full spectra confirm the presence of C, O, and N elements in the hydrogel (Fig. 1b_1_; Fig. S2a_1_–c_1_), with N signals verifying the successful dispersion of BBR (Fig. S2 d_1_–e_1_). High-resolution C 1s spectra of PVA/BBR/HD-4 (Fig. 1b_2_) reveal peaks corresponding to C–N (287.3 eV), C–O (286.1 eV), C–C/C–H (284.6 eV), and C=N (283.1 eV), while the O 1s spectrum (Fig. 1b3) indicates dominant C–O (531.1 eV) and O–H (532.3 eV) contributions. N 1s spectra (Fig. 1b4) show peaks at 399.8 eV and 401.2 eV, assigned to C–N–C and N=C, respectively [[44], [45], [46]]. Hydrogels with varying compositions all exhibited characteristic peaks consistent with those described above, and no new chemical bonding peaks were observed when compared to the XPS spectra of pure BBR and HD (Fig. S2 a_2–4_, b_2–4_, c_2–4_, d_2-4_, e_2-3_), indicating that PVA, BBR, and HD primarily form a physically cross-linked network through hydrogen bonding rather than chemical reactions [47]. Collectively, these results confirm the successful fabrication of the PVA/BBR/HD hydrogel.

Scanning electron microscopy (SEM) images reveal distinct microstructural differences among the hydrogels (Fig. 1c). Compared to the PVA/BBR hydrogel, which exhibits larger pores and a relatively loose network, the incorporation of HD progressively reduces pore size and increases internal density, decreasing the average surface pore diameter from 21.5 μm to 10.56 μm. This effect is attributed to HD's polyhydroxy structure, which enhances hydrogen bonding and crosslinking density within the hydrogel network. The resulting moderately porous structure supports cell infiltration, adhesion, and nutrient exchange, making it suitable for wound dressing applications [48]. However, excessively small pores may increase diffusion resistance, potentially impairing drug release efficiency.

The mechanical properties of the hydrogels were evaluated through compression testing (Fig. 1d and e). As the HD content increased, the compressive strength of the hydrogels significantly improved. At 80% strain, the PVA/BBR hydrogel exhibited a compressive strength of 35.74 ± 3.77 kPa, whereas the PVA/BBR/HD-6 hydrogel reached 102.64 ± 3.22 kPa, demonstrating that HD incorporation effectively enhanced the mechanical stability of the hydrogels. Additionally, the hydrogels displayed excellent elasticity and deformation recovery, meeting the mechanical requirements for various wound applications. Swelling behavior, a critical parameter for evaluating hydrogel wound dressings, was assessed at pH 7.4 (Fig. 1f) [49]. The PVA/BBR hydrogel reached swelling equilibrium within approximately 5 h, whereas the PVA/BBR/HD hydrogels required about 8 h. After 24 h, the swelling ratios of the PVA/BBR, PVA/BBR/HD-2, PVA/BBR/HD-4, and PVA/BBR/HD-6 hydrogels were 173.51 ± 7.52%, 261.93 ± 1.22%, 430.00 ± 12.93%, and 566.84 ± 12.33%, respectively. The PVA/BBR/HD-4 hydrogel exhibited moderate swelling, providing an optimal moist environment for wound repair, whereas excessive swelling may increase the risk of tissue compression [50,51]. *In vitro* degradation studies (Fig. 1g) demonstrated a gradual decrease in the degradation rate with increasing HD content, creating a gradient from rapid to slow degradation. After 7 days of incubation in PBS, the PVA/BBR/HD-4 hydrogel exhibited a mass loss of approximately 29.46%, indicating a moderate degradation rate favorable for new tissue growth and integration [52].

Rheological characterization further elucidated the viscoelastic properties of the hydrogels. Frequency sweeps conducted at 1% strain over a range of 0.01 to 100 Hz demonstrated that the storage modulus (G′) values of PVA/BBR/HD-4 and PVA/BBR/HD-6 remained higher than the loss modulus (G″), whereas PVA/BBR and PVA/BBR/HD-2 exhibited frequency-dependent variations, indicating insufficient modulus for mechanical stability (Fig. 1h_1_, Fig. S3 a_1_). Alternating step-strain tests (Fig. 1h_2_, Fig. S3 a_2_) showed that the PVA/BBR/HD-4 hydrogel exhibited reversible changes in G′ and G″ under low and high strains, reflecting excellent self-healing capability. In contrast, PVA/BBR and PVA/BBR/HD-2 hydrogels demonstrated weaker reversibility, while PVA/BBR/HD-6 displayed a limited sol–gel transition due to excessive HD content. Within the temperature range of 25–45 °C, PVA/BBR/HD-4 maintained stable elastic properties (Fig. 1h_3_, Fig. S3 a_3_), making it suitable for slightly elevated wound microenvironments. Shear viscosity tests showed that the PVA/BBR/HD-6 hydrogel exhibited the highest viscosity (Fig. 1h_4,_ Fig. S3 a4). Due to its superior mechanical properties, the injection force required for the PVA/BBR/HD-6 hydrogel was significantly higher than that for the PVA/BBR/HD-4 hydrogel, reaching approximately 36.88 N. In contrast, the injection force for the PVA/BBR/HD-4 hydrogel was approximately 4.54 N (Fig. S4), which is below the clinically recommended upper limit of 20 N [53]. Considering mechanical properties, rheological behavior, and injectability, PVA/BBR/HD-4 was selected as the optimal formulation for subsequent studies.

The shear-thinning injectability of the PVA/BBR/HD-4 hydrogel is illustrated in Fig. 1i_1_–i_2_. Under shear stress, the hydrogel transitions from a gel to a sol state, enabling injection, and subsequently self-heals to reform the gel within 22 min, as confirmed by the inverted tube method. This behavior is primarily attributed to hydrogen bonds, which, although individually weak, collectively provide covalent-like reinforcement [54]. Similar self-healing supramolecular hydrogels have been reported by Das et al. and Watanabe et al. [55,56]. Notably, the PVA/BBR/HD-4 hydrogel can be extruded to draw irregular patterns without clogging (Fig. 1j). When injected into irregular silicone molds, it uniformly fills the mold within 5 min, forming complex shapes (Fig. 1k). These results confirm the self-healing properties of the PVA/BBR/HD-4 hydrogel and its ability to conform to the irregular shape of the wound. This characteristic is crucial for ensuring complete filling of the tissue defect, maintaining the hydrogel's structural integrity, and enabling controlled drug release without causing damage [57].

### 2.2 Real-time *in situ* pH monitoring using the PVA/BBR/HD hydrogel

**Fig. 2:**
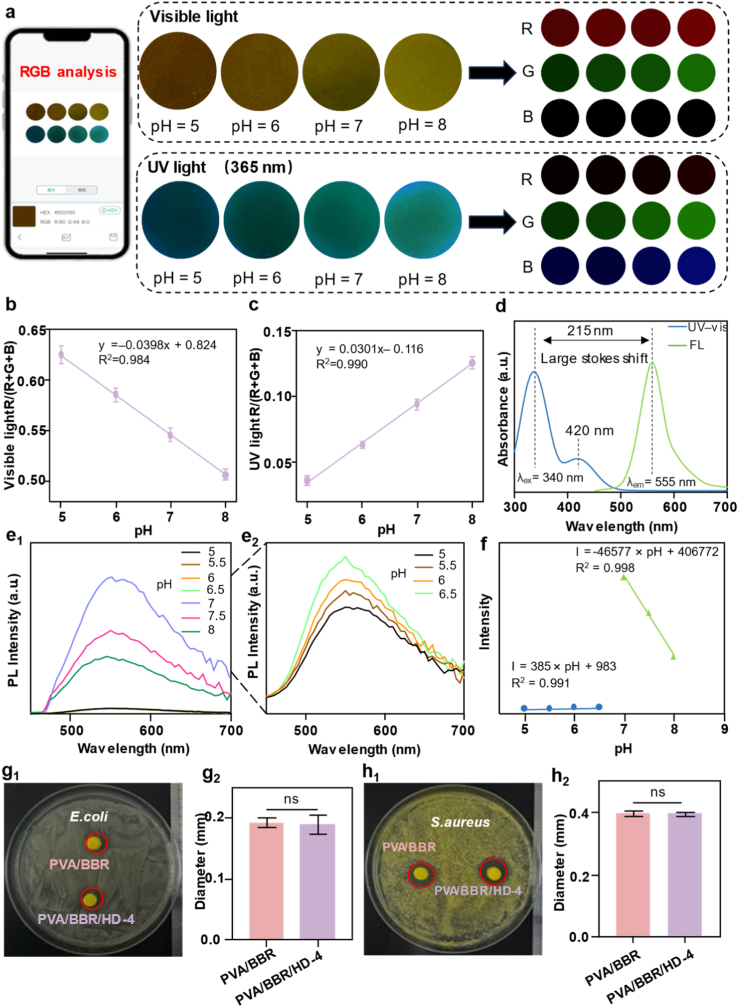
Optical properties of PVA/BBR/HD hydrogels. (a) Smartphone-assisted real-time pH monitoring capability of the PVA/BBR/HD-4 hydrogel. Variation of R/(R + G + B) values as a function of pH under (b) visible light and (c) UV irradiation (n = 3). (d) UV–vis absorption and fluorescence emission spectra. Photoluminescence (PL) spectra of the PVA/BBR/HD-4 hydrogel at different pH ranges: (e_1_) 5–8 and (e_2_) 4.5–6.5. (f) PL intensity at λ_em_ = 575 nm as a function of pH. Antibacterial activity images and quantitative analysis of the PVA/BBR/HD-4 hydrogel against (g_1–2_) *E. coli* and (g_3–4_) *S. aureus* (n = 3). Statistical significance was assessed using Student's t-test; ns indicates no significant difference.

Dynamic pH fluctuations within the wound microenvironment serve as critical indicators of tissue repair progression and infection risk. Therefore, real-time, non-invasive monitoring of local wound pH is essential for guiding precise therapeutic interventions. Based on the PVA/BBR/HD-4 hydrogel, we developed an integrated *in situ* sensing system that combines dual-mode colorimetric and fluorescent detection. The PVA/BBR/HD-4 hydrogel responds to pH changes within 1 min and, when paired with a smartphone, enables real-time visual monitoring of wound pH in under 1 s. As illustrated in Fig. 2a, the entire pH detection process was conducted using smartphone-based image acquisition under both visible light and fluorescence modes. As the pH increases from acidic to alkaline conditions, the hydrogel color gradually changes from dark brown to yellow under visible light, enabling intuitive visual assessment of wound status. Under UV excitation, the fluorescence color intensifies from dark green to bright green, providing an enhanced fluorescent response to pH variations. To enable quantitative pH discrimination, RGB values were extracted from hydrogel images using smartphone-based color recognition software under both optical modes, and standard color charts corresponding to different pH levels were established. Further calibration of the relationship between RGB intensity and pH significantly improved resistance to external interference and enhanced detection accuracy. As shown in Fig. 2b, under visible-light conditions, the R/(R + G + B) value decreases progressively with increasing pH, with a detection limit of 0.3 pH units. In contrast, under UV excitation, the R/(R + G + B) value increases monotonically with rising pH, with a detection limit of 0.5 pH units (Fig. 2c), demonstrating complementary pH-responsive behaviors across the two optical modes.

To further elucidate the optical properties of the PVA/BBR/HD-4 hydrogel, UV–vis absorption and fluorescence spectroscopy were conducted. As shown in Fig. 2d, the hydrogel exhibits a prominent absorption peak at 340 nm and a weaker absorption band near 420 nm. The corresponding fluorescence emission peak is centered at 555 nm, resulting in a large Stokes shift of approximately 215 nm. This substantial Stokes shift effectively minimizes self-absorption and background fluorescence interference, making the hydrogel particularly suitable for portable optical sensing and bioimaging applications [58]. Comparative photoluminescence (PL) analysis under different pH conditions (Fig. 2e_1_–e_2_) revealed that the PVA/BBR/HD-4 hydrogel exhibited maximal fluorescence intensity at neutral pH (7.0). In contrast, under acidic or alkaline conditions, the fluorescence intensity decreased significantly and was accompanied by a pronounced red shift. Notably, the fluorescence intensity showed a strong linear correlation with pH over the range of pH 5–8 (Fig. 2f). This responsive window precisely encompasses the pH variations characteristic of both normal and pathological wound microenvironments, underscoring its practical utility for wound status monitoring and treatment guidance.

To verify the repeatability of pH monitoring, independently prepared hydrogel samples were used for continuous measurements at three time points (morning, noon, and evening) under indoor visible light and UV light conditions (Fig. S5). The results showed that the relative standard deviation of the pH values obtained through RGB conversion was less than 0.3%, indicating that the sensing system exhibits excellent stability and repeatability. Assess the long-term pH monitoring performance of the PVA/BBR/HD hydrogel in a physiologically relevant environment, a standard mouse wound model was employed to continuously monitor the hydrogel's colorimetric response over a 72 h period (Fig. S6). The results demonstrated that the pH values measured by the PVA/BBR/HD-4 hydrogel closely corresponded with those obtained using pH indicator strips, indicating that this system provides reliable pH sensing capabilities in an *in vivo*–relevant environment.

In addition, the antibacterial activities of PVA/BBR and PVA/BBR/HD-4 hydrogels were evaluated against *Escherichia coli* (*E. coli*) and *Staphylococcus aureus* (*S. aureus*) using zone-of-inhibition assays (Fig. 2g and h). No significant differences in inhibition zone diameters were observed between the two hydrogel formulations, indicating that the incorporation of HD did not compromise the intrinsic antimicrobial activity of BBR. These results confirm that the PVA/BBR/HD-4 hydrogel retains robust antibacterial efficacy while achieving multifunctional integration.

The release behavior of BBR from the PVA/BBR/HD-4 hydrogel was further evaluated (Fig. S7a_1_). At 37 °C, the cumulative release was 8.21 ± 1.07% after 1 h, increasing to 26.75 ± 0.93% after 24 h. These results indicate that the hydrogel exhibits favorable sustained-release properties, providing a material basis for prolonged antimicrobial activity. Subsequently, antimicrobial kinetics were systematically assessed through time-dependent killing assays. The results demonstrated that at higher concentrations, bacterial counts decreased significantly within a few hours, whereas the control group showed a gradual increase over time. Notably, both the 2 × MIC and 4 × MIC groups effectively reduced bacterial counts, with a more pronounced inhibitory effect observed against *S. aureus* (Fig. S7a_2–3_).

In summary, the PVA/BBR/HD-4 hydrogel not only retains the antibacterial activity of BBR but also provides sustained and rapid antibacterial effects through its controlled release, demonstrating a clear concentration-dependent response.

### 2.3 Biocompatibility

To assess the biosafety of the PVA/BBR/HD-4 hydrogel for skin wound repair applications, its cytotoxicity, hemocompatibility, and *in vivo* host response were systematically evaluated. After exposure to PVA/BBR/HD-4 hydrogel extracts at various concentrations, the viability of L929 fibroblasts showed no significant difference compared to the control group (Fig. S8), indicating negligible cytotoxicity. Hemolysis assays further revealed a hemolysis rate of approximately 0.55%, well below the accepted safety threshold of 5% (Fig. S9), confirming the hydrogel's excellent hemocompatibility and suitability for biomedical use [59].

*In vivo* biocompatibility was further evaluated through subcutaneous implantation studies. Compared to the control group, no significant changes were observed in routine blood parameters in mice implanted with the PVA/BBR/HD-4 hydrogel (Table S2). As the hydrogel gradually degraded and was absorbed by the host, it became macroscopically undetectable, and no visible signs of congestion, erythema, or inflammatory responses were observed at the implantation site (Fig. S10). Histological examination revealed minimal inflammatory cell infiltration in hematoxylin and eosin (HE)-stained sections of the implantation region. Furthermore, no pathological damage or abnormal structural changes were detected in major organs, including the heart, liver, spleen, lungs, and kidneys (Fig. S11).

Collectively, these findings demonstrate that the PVA/BBR/HD-4 hydrogel exhibits excellent biocompatibility both *in vitro* and *in vivo*, without causing any noticeable acute or chronic toxicity. These results provide a strong safety foundation for its further application in skin wound repair and regenerative medicine.

### 2.4 *In vitr*o evaluation of the PVA/BBR/HD hydrogel activity

**Fig. 3:**
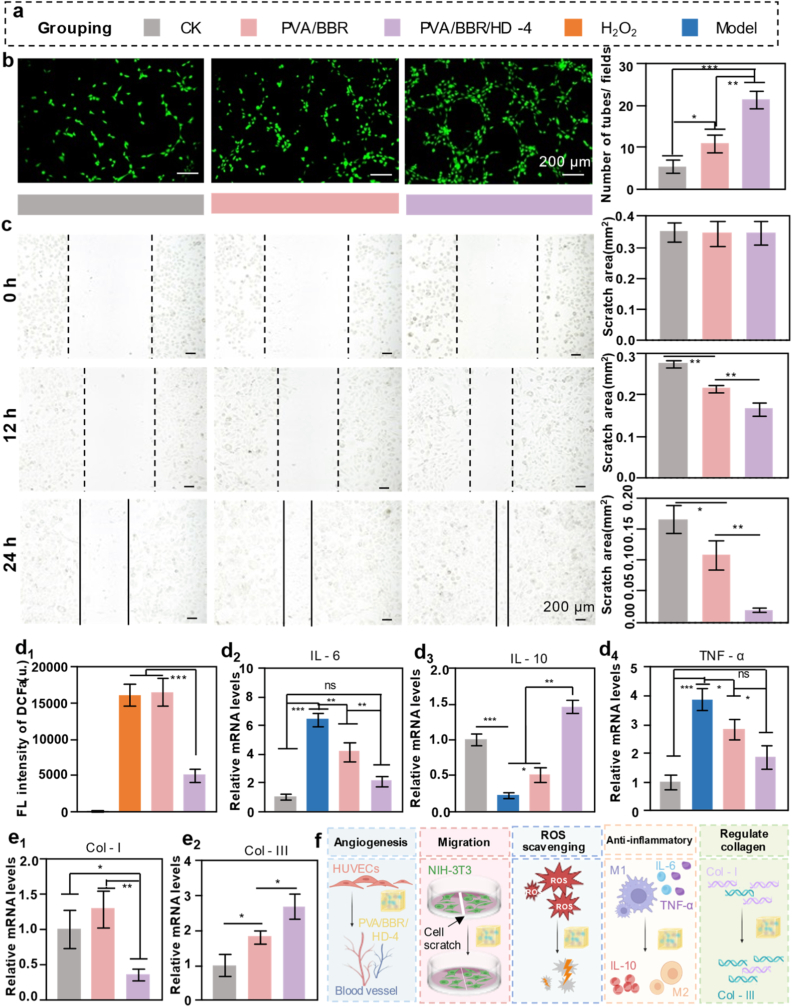
*In vitro* biological effects of PVA/BBR/HD-4 hydrogels. (a) Schematic illustration of the experimental groups. (b) HUVEC tube formation assay and quantitative analysis following treatment with PVA/BBR and PVA/BBR/HD-4 hydrogels (n = 3). (c) Cell migration assay and quantitative analysis of NIH-3T3 fibroblasts (n = 3). (d_1_) Intracellular ROS scavenging ability of PVA/BBR and PVA/BBR/HD-4 hydrogels in RAW 264.7 cells (n = 3). Expression of inflammation-related genes in RAW 264.7 cells treated with PVA/BBR and PVA/BBR/HD-4 hydrogels: (d_2_) IL-6, (d_3_) IL-10, and (d_4_) TNF-α. Expression of collagen-related genes in L929 cells: (e_1_) Col I and (e_2_) Col III (n = 3). (f) Proposed mechanism underlying PVA/BBR/HD-4 hydrogel–mediated scarless wound healing. Statistical significance was analyzed using one-way ANOVA (∗*p*< 0.05, ∗∗*p*< 0.01, ∗∗∗*p* < 0.001), ns indicates no significant difference. Error bars represent standard deviation.

The proliferative phase of wound healing is a critical stage for tissue reconstruction, involving epithelial regeneration, angiogenesis, and ECM deposition and remodeling. The experimental design is summarized in Fig. 3a. Angiogenesis assays demonstrated that, compared to the control and PVA/BBR groups, the PVA/BBR/HD-4 hydrogel significantly enhanced the *in vitro* tube-forming ability of human umbilical vein endothelial cells (HUVECs) (Fig. 3b), indicating a strong pro-angiogenic effect. Neovascularization is essential for delivering oxygen and nutrients to regenerating tissues and is a key prerequisite for accelerated wound repair. Fibroblast migration, a key feature of the proliferative phase, was assessed using a scratch assay (Fig. 3c). Quantitative analysis of wound closure at 0, 12, and 24 h revealed that both PVA/BBR and PVA/BBR/HD-4 hydrogels significantly enhanced NIH-3T3 fibroblast migration compared to the control group, with the PVA/BBR/HD-4 hydrogel showing the most pronounced effect. These findings indicate that the incorporation of HD plays a crucial role in promoting fibroblast motility, thereby conferring superior repair-enhancing properties to the composite hydrogel compared to the single-component BBR system.

In diabetic wound microenvironments, hyperglycemia-induced mitochondrial dysfunction—particularly abnormalities in the respiratory chain—leads to excessive intracellular accumulation of ROS, triggering oxidative stress and severely impairing tissue repair. To evaluate the antioxidant capacity of the hydrogels, intracellular ROS levels in RAW 264.7 macrophages were measured using DCFH-DA probes. As shown in Fig. 3d_1_, H_2_O_2_ stimulation markedly increased ROS levels in the model group. While the PVA/BBR group showed no significant reduction compared to the model group, treatment with the PVA/BBR/HD-4 hydrogel substantially decreased intracellular ROS levels, as evidenced by significantly reduced fluorescence intensity, indicating effective mitigation of oxidative stress.

Given the central role of macrophage-mediated inflammation in chronic and diabetic wounds, the anti-inflammatory activity of the PVA/BBR/HD-4 hydrogel was further investigated. As shown in Fig. 3d_2–4_, reverse transcription polymerase chain reaction (RT-PCR) analysis revealed that treatment with the PVA/BBR/HD-4 hydrogel significantly downregulated the expression of pro-inflammatory cytokines (IL-6 and TNF-α) while markedly upregulating the anti-inflammatory cytokine IL-10 in RAW 264.7 cells. Notably, pro-inflammatory gene expression levels in the PVA/BBR/HD-4 group were comparable to those in the control group and significantly lower than those in the model and PVA/BBR groups. These results indicate that HD incorporation substantially enhances the immunomodulatory capacity of the hydrogel, promoting macrophage polarization toward the M2 phenotype and facilitating the resolution of excessive inflammation. Collagen composition critically influences scar formation during wound healing. Previous studies have demonstrated that fetal wounds heal without scarring, a phenomenon largely attributed to a higher proportion of Col III relative to Col I [60]. Consistent with this paradigm, the PVA/BBR/HD-4 hydrogel significantly increased Col III expression while concurrently suppressing Col I expression in cultured fibroblasts (Fig. 3e_1–2_), suggesting a favorable remodeling of collagen composition toward a scar-minimizing phenotype. Collectively, these results demonstrate that the PVA/BBR/HD-4 hydrogel synergistically promotes angiogenesis and fibroblast migration, effectively alleviates oxidative stress, modulates the inflammatory microenvironment, and optimizes collagen composition. Through these coordinated biological effects, the hydrogel exhibits comprehensive *in vitro* advantages for promoting functional, scar-free wound healing (Fig. 3f).

### 2.5 *In vivo* antibacterial activity and wound healing

**Fig. 4:**
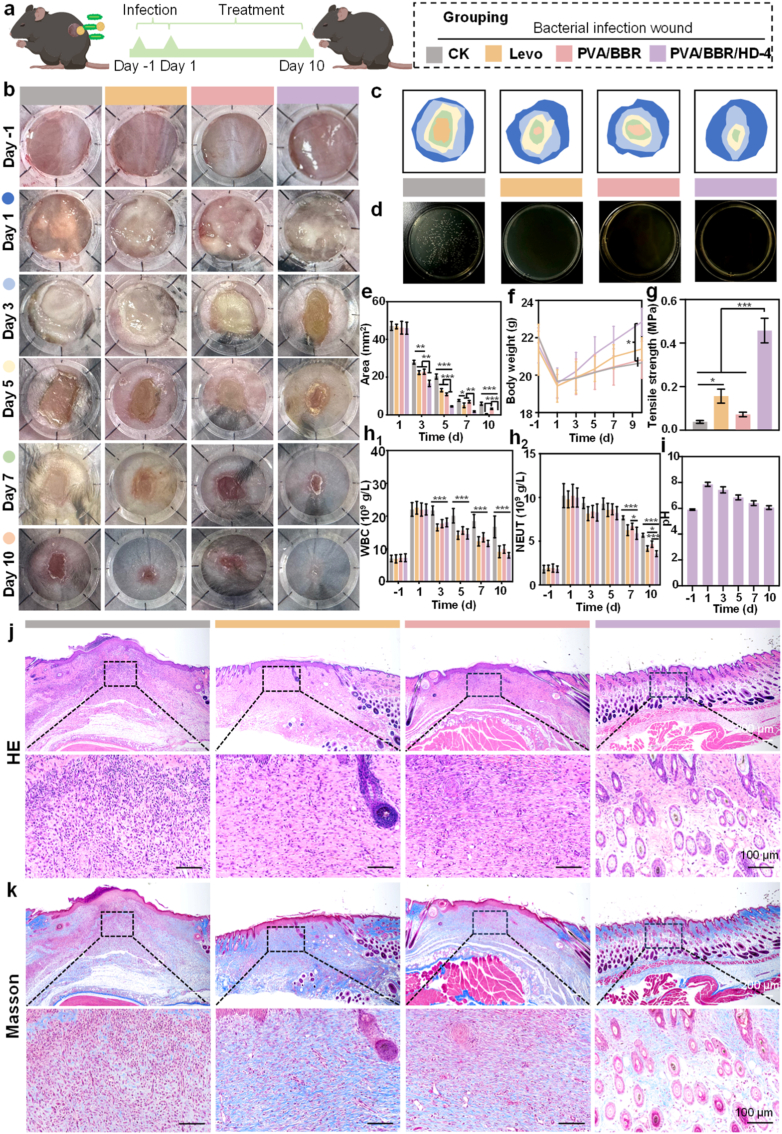
*In vivo* evaluation of the PVA/BBR/HD-4 hydrogel in a bacteria-infected wound model. (a) Schematic illustration of the mixed *S. aureus* and *E. coli* infected wound model and treatment timeline. (b) Representative wound photographs. (c) Schematic representation of wound closure. (d) Bacterial colony counts from wound tissues after treatment. (e) Quantification of wound area and (f) body weight changes during the healing process in the control (PBS), positive control (levofloxacin hydrochloride), and experimental groups (PVA/BBR and PVA/BBR/HD-4 hydrogels) (n = 6). (g) Tensile testing of healed skin tissues (n = 3). (h_1_) White blood cell (WBC) counts and (h_2_) neutrophil (NEUT) levels during wound healing (n = 6). (i) pH variation at infected wound sites treated with the PVA/BBR/HD-4 hydrogel (n = 6). Statistical significance was analyzed using one-way ANOVA (∗*p*< 0.05, ∗∗*p*< 0.01, ∗∗∗*p* < 0.001). Error bars represent standard deviation.

Bacterial infection, particularly mixed bacterial infections, represents a significant obstacle to chronic wound healing and is a primary cause of delayed repair and treatment failure. To evaluate the *in vivo* antibacterial efficacy and wound-healing potential of the PVA/BBR/HD-4 hydrogel, a full-thickness dorsal skin wound model infected with a mixed culture of *S. aureus* and *E. coli* was established in mice (Fig. 4a). As shown in Fig. 4b and c, pronounced purulent exudation was observed at the 8 mm full-thickness wound sites after 48 h of bacterial exposure, confirming the successful establishment of the infected wound model. Wound areas progressively decreased in all groups throughout the treatment period. Notably, by day 3, scab formation was already evident in wounds treated with the PVA/BBR/HD-4 hydrogel, whereas substantial exudation persisted in the control group. By day 10, wounds treated with the PVA/BBR/HD-4 hydrogel were nearly completely healed, exhibiting skin that closely resembled normal tissue, characterized by a smooth epidermis, uniform coloration, and favorable elasticity. In contrast, wounds in the control and PVA/BBR groups remained partially open, while those treated with levofloxacin (Levo) showed prominent scar formation. Bacterial burden analysis further confirmed the antibacterial efficacy of the hydrogel (Fig. 4d). Abundant bacterial colonies were detected in wound tissues from the control group, whereas bacterial growth was markedly suppressed in the PVA/BBR/HD-4, PVA/BBR, and Levo groups. Quantitative analysis of wound closure (Fig. 4e) showed no significant differences among groups before treatment initiation. However, throughout the treatment period, wounds in the PVA/BBR/HD-4 group closed significantly faster than those in all other groups, highlighting its superior therapeutic efficacy in infected wound repair. Importantly, all animals exhibited stable weight gain and a 100% survival rate during treatment (Fig. 4f), further confirming the favorable *in vivo* biosafety profile of the PVA/BBR/HD-4 hydrogel.

To evaluate the quality and functional integrity of the regenerated tissue, uniaxial tensile testing was conducted on healed skin samples. The PVA/BBR/HD-4 group demonstrated significantly higher tensile strength compared to all other groups, while no significant difference was observed between the PVA/BBR and control groups (Fig. 4g). These findings suggest that HD plays a crucial role in enhancing tissue extensibility and mechanical strength, thereby contributing to improved functional recovery and reduced scarring. Systemic inflammatory responses were assessed by measuring white blood cell (WBC) and neutrophil (NEUT) counts (Fig. 4h_1–2_). At 48 h post-infection, both parameters were elevated across all groups. With continued treatment, WBC and NEUT levels gradually decreased in the PVA/BBR/HD-4, PVA/BBR, and Levo groups, whereas persistently elevated levels were observed in the control group. Concurrently, real-time monitoring of local wound pH in the PVA/BBR/HD-4 group showed an initial pH increase during infection, followed by a gradual decrease as healing progressed, ultimately returning to near-physiological levels by day 10 (Fig. 4i). This dynamic pH trajectory underscores the hydrogel's capability for real-time, *in situ* monitoring of wound status and highlights its potential for precision wound management.

Wound repair quality is commonly evaluated based on wound closure, epithelial regeneration, and the reconstruction of skin appendages such as hair follicles and glands [61]. HE and Masson's trichrome staining (Fig. 4j and k) demonstrated pronounced inflammatory cell infiltration, abnormal thickening of the keratin layer, and incomplete epithelialization in the control group. The PVA/BBR and Levo groups also exhibited varying degrees of residual inflammation and limited hair follicle regeneration. In contrast, wounds treated with the PVA/BBR/HD-4 hydrogel showed markedly accelerated epithelial regeneration, robust reconstruction of hair follicles and skin appendages, and well-organized collagen deposition within 14 days. Overall, the PVA/BBR/HD-4 hydrogel demonstrated antibacterial efficacy comparable to that of Levo, while facilitating higher-quality tissue regeneration that more closely mimics the structure and mechanical properties of native skin. By simultaneously suppressing infection, modulating inflammation, and promoting organized tissue reconstruction, the hydrogel effectively supports functional, scar-minimized wound healing.

### 2.6 *In vivo* burn wound healing

**Fig. 5:**
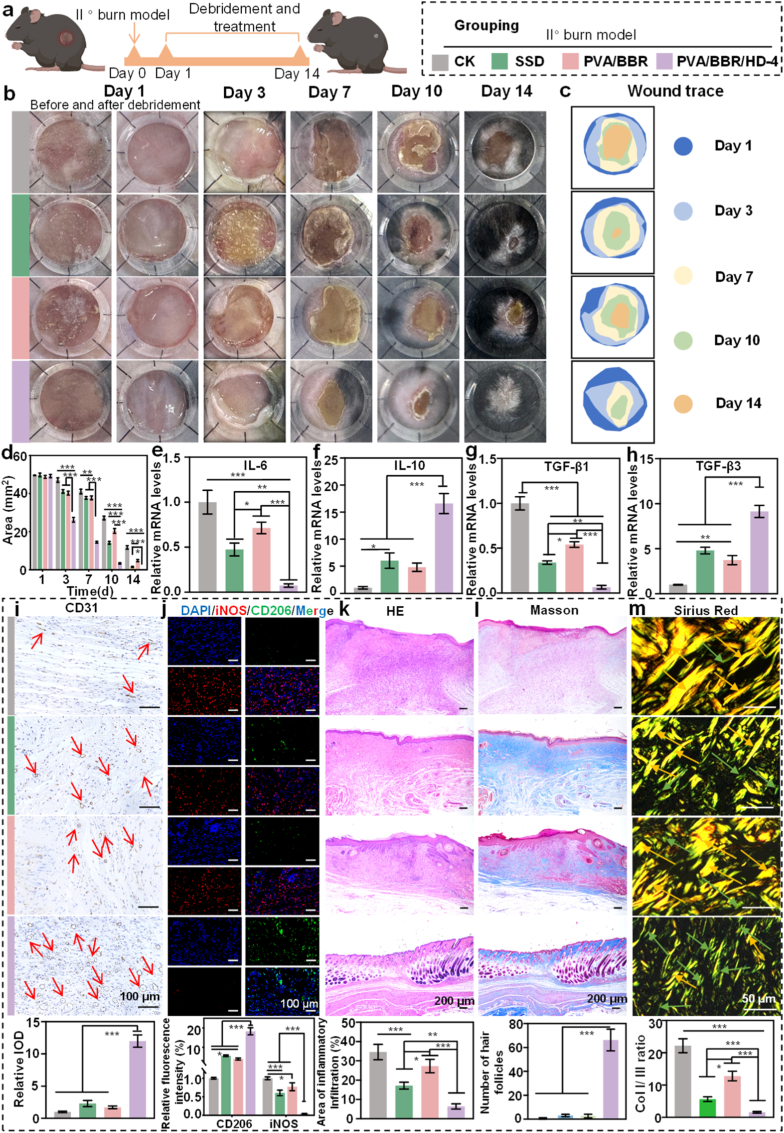
*In vivo* evaluation of the PVA/BBR/HD-4 hydrogel in a burn wound model. (a) Schematic illustration of the establishment of a second-degree burn model and the treatment timeline. (b) Representative wound photographs. (c) Wound closure traces and (d) quantitative analysis of wound area in different treatment groups (n = 6). On day 7, mRNA expression levels of (e) IL-6, (f) IL-10, (g) TGF-β1, and (h) TGF-β3 in wound tissues were analyzed by real-time PCR (n = 3). Representative images of (i) CD31 immunohistochemical staining and (j) immunofluorescence staining of wound tissues, along with corresponding quantitative analyses on day 7. Representative images of (k) HE, (l) Masson's trichrome, and (m) Sirius Red staining, along with corresponding quantitative analyses on day 14. Statistical significance was determined using one-way ANOVA (∗*p*< 0.05, ∗∗*p*< 0.01, ∗∗∗*p* < 0.001). Error bars represent standard deviation. (For interpretation of the references to color in this figure legend, the reader is referred to the Web version of this article.)

Burn injuries are a common and severe form of skin damage. Second-degree or more severe burns typically trigger intense inflammatory responses and pronounced proliferative scar formation [62]. To assess the therapeutic efficacy of the PVA/BBR/HD-4 hydrogel in burn wound repair, a murine second-degree burn chronic wound model was established (Fig. 5a). Wound healing progression was continuously monitored from day 0 to day 14 across different treatment groups (Fig. 5b and c). Macroscopic observations revealed that wounds treated with the PVA/BBR/HD-4 hydrogel exhibited rapid contraction and closure, achieving near-complete healing by day 14, which was markedly superior to the other treatment groups. In contrast, wounds in the control group remained substantially larger and showed a significantly delayed healing process. Quantitative analysis of wound area (Fig. 5d) further confirmed that the PVA/BBR/HD-4 group maintained significantly smaller wound areas than all other groups throughout the entire treatment period, indicating its robust therapeutic efficacy in burn wound repair. To elucidate the inflammatory microenvironment during burn healing, inflammatory cytokine expression in wound tissues was analyzed on day 7 post-treatment. As shown in Fig. 5e and f, the expression of the pro-inflammatory cytokine IL-6 was significantly elevated in the control group, whereas treatment with the PVA/BBR/HD-4 hydrogel markedly suppressed IL-6 expression. Conversely, the anti-inflammatory cytokine IL-10 was significantly upregulated in the PVA/BBR/HD-4 group, indicating effective modulation of the local inflammatory response. Furthermore, analysis of fibrosis-related factors demonstrated that the PVA/BBR/HD-4 group exhibited significantly reduced relative mRNA expression of TGF-β1 and a concomitant increase in TGF-β3 expression, resulting in a markedly elevated TGF-β3/TGF-β1 ratio compared with the other groups (Fig. 5g and h). This shift in the TGF-β isoform balance is closely associated with the suppression of post-burn fibrosis and hypertrophic scar formation [63]. Additionally, dynamic monitoring revealed that local wound pH gradually decreased from the initial alkaline state during infection and returned to the physiological range by day 14 (Fig. S12).

Wound healing is a highly coordinated, multistep biological process involving wound contraction, granulation tissue formation, scar development, and epidermal regeneration [64]. To further elucidate the underlying mechanisms of tissue repair, wound tissues were harvested on days 7 and 14 for comprehensive histological evaluation. Angiogenesis is a critical determinant of effective wound healing, and impaired neovascularization is known to significantly hinder burn wound recovery [65]. CD31 immunohistochemical staining on day 7 revealed the highest microvessel density in the PVA/BBR/HD-4 group (Fig. 5i), indicating enhanced angiogenic activity. Macrophage polarization analysis further demonstrated that treatment with the PVA/BBR/HD-4 hydrogel significantly promoted macrophage polarization toward the anti-inflammatory M2 phenotype, as evidenced by the downregulation of the pro-inflammatory marker iNOS and the upregulation of the M2-associated marker CD206 (Fig. 5j). This phenotypic shift contributes to the effective resolution of inflammation and supports tissue regeneration.

By day 14, histological analysis revealed that wounds treated with the PVA/BBR/HD-4 hydrogel exhibited a thinner yet structurally intact dermis, a smooth and continuous epidermal layer, and a markedly reduced area of inflammatory cell infiltration (Fig. 5k). Masson's trichrome and Sirius red staining further confirmed more orderly collagen deposition, a significantly reduced Col I/Col III ratio, and a pronounced increase in skin appendage regeneration in this group (Fig. 5l and m). Collectively, these results demonstrate that the PVA/BBR/HD-4 hydrogel facilitates high-quality, scar-minimized repair of burn wounds by synergistically suppressing excessive inflammation and fibrosis while promoting angiogenesis, re-epithelialization, and functional tissue remodeling.

### 2.7 Wound healing in diabetes

**Fig. 6:**
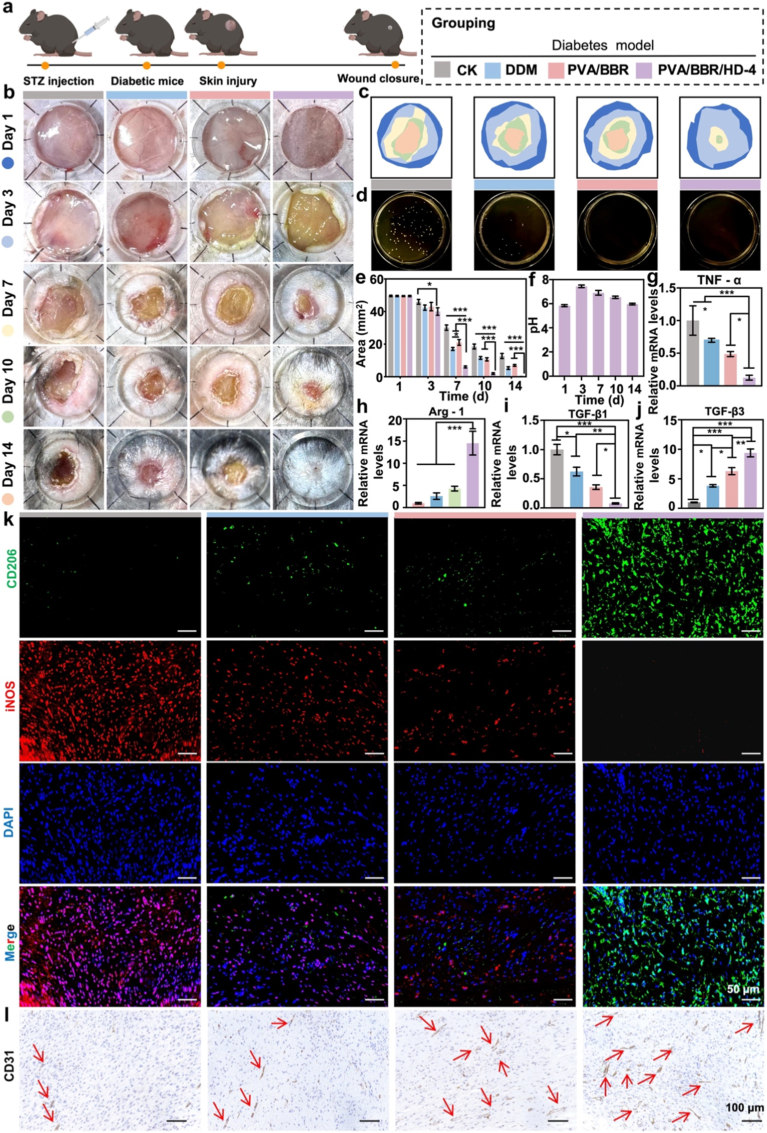
PVA/BBR/HD-4 hydrogel accelerates full-thickness diabetic wound healing in mice. (a) Schematic illustration of PVA/BBR/HD-4 hydrogel application in diabetic wound healing. (b) Representative wound photographs. (c) Schematic representation of wound closure progression. (d) Bacterial colony counts from wound tissues after treatment. (e) Quantification of wound area during healing in different groups (n = 6). (f) pH variation at the diabetic wound sites treated with PVA/BBR/HD-4 hydrogel (n = 6). On day 7, mRNA expression levels of (g) TNF-α, (h) Arg-1, (i) TGF-β1, and (j) TGF-β3 were quantified by real-time PCR (n = 3). Representative images of (k) immunofluorescence staining and (l) CD31 immunohistochemical staining of wound tissues on day 7. Statistical significance was analyzed using one-way ANOVA (∗*p*< 0.05, ∗∗*p*< 0.01, ∗∗∗*p* < 0.001). Error bars represent standard deviation.

Unlike acute injuries such as burns, diabetic wounds persist within a chronic pathological microenvironment characterized by increased susceptibility to infection, biofilm formation, sustained inflammatory activation, impaired vascular integrity, and reduced tissue remodeling capacity. This complex interplay of metabolic dysregulation and immune dysfunction severely impairs spontaneous wound healing, making diabetic infected wounds (DIW) a significant clinical challenge [66,67]. To assess the therapeutic potential of the PVA/BBR/HD-4 hydrogel under these highly adverse conditions, an infected full-thickness skin defect model with a diameter of 0.8 cm was created on the dorsal region of diabetic mice (Fig. 6a). Macroscopic assessment revealed that wounds treated with the PVA/BBR/HD-4 hydrogel consistently exhibited more rapid contraction throughout the healing process compared to other groups (Fig. 6b). Dynamic heatmaps generated from wound area measurements further demonstrated a more efficient and continuous healing trajectory at all critical time points (Fig. 6c), indicating sustained therapeutic efficacy of the hydrogel within the diabetic microenvironment. Given that infection is a principal factor impeding diabetic wound repair, bacterial burden in wound tissue and exudate was systematically quantified. Both BBR-containing treatment groups significantly reduced local bacterial colonization, confirming the sustained *in vivo* antibacterial activity of BBR (Fig. 6d). Notably, by day 14 of treatment, the residual wound area in the PVA/BBR/HD-4 group decreased to approximately 0.1 mm^2^, which was significantly smaller than those observed in the control, DuoDerm™ hydrogels (DDM), and PVA/BBR groups (Fig. 6e), demonstrating a clear advantage in promoting tissue repair beyond infection control alone. As shown in Fig. 6b, the initial wound pH in the PVA/BBR/HD-4-treated group was approximately 5.84 and increased during the infection and inflammatory phases. With progressive wound healing, the local pH gradually decreased, returning to approximately 6.0 by day 14, approaching the physiological pH range of healthy skin. This dynamic trend reflects the stabilization of the wound microenvironment and progression toward complete healing (Fig. 6f).

At the molecular level, DIW are typically characterized by persistent inflammatory activation. Analysis conducted on day 7 revealed that the expression of the pro-inflammatory cytokine TNF-α remained elevated in the control group, whereas it was significantly reduced following treatment with the PVA/BBR/HD-4 hydrogel (Fig. 6g). Concurrently, the expression of the anti-inflammatory marker Arg-1 was markedly upregulated (Fig. 6h), indicating a shift in the local inflammatory state from sustained activation toward resolution. Moreover, PVA/BBR/HD-4 treatment significantly downregulated TGF-β1 expression while simultaneously increasing TGF-β3 levels (Fig. 6i and j), suggesting effective suppression of aberrant fibrotic responses under diabetic conditions. Immunofluorescence staining was used to further evaluate macrophage phenotype modulation (Fig. 6k, Fig. S13). Compared to the untreated control group, all treatment groups demonstrated varying degrees of immunomodulatory effects and partial alleviation of inflammation. Notably, the PVA/BBR/HD-4 hydrogel induced the most pronounced increase in the anti-inflammatory marker CD206, while significantly suppressing the expression of the pro-inflammatory marker iNOS. These findings indicate that the hydrogel effectively directs macrophage polarization toward a repair-associated M2 phenotype, thereby creating a more favorable immune microenvironment for diabetic wound regeneration. Impaired angiogenesis is a critical limiting factor in diabetic wound healing. CD31 immunohistochemical analysis demonstrated that by day 7, the PVA/BBR/HD-4 group exhibited significantly higher neovascular density within the wound area compared to the other groups (Fig. 6l, Fig. S14), highlighting its distinct advantage in promoting vascular regeneration under diabetic conditions.

**Fig. 7:**
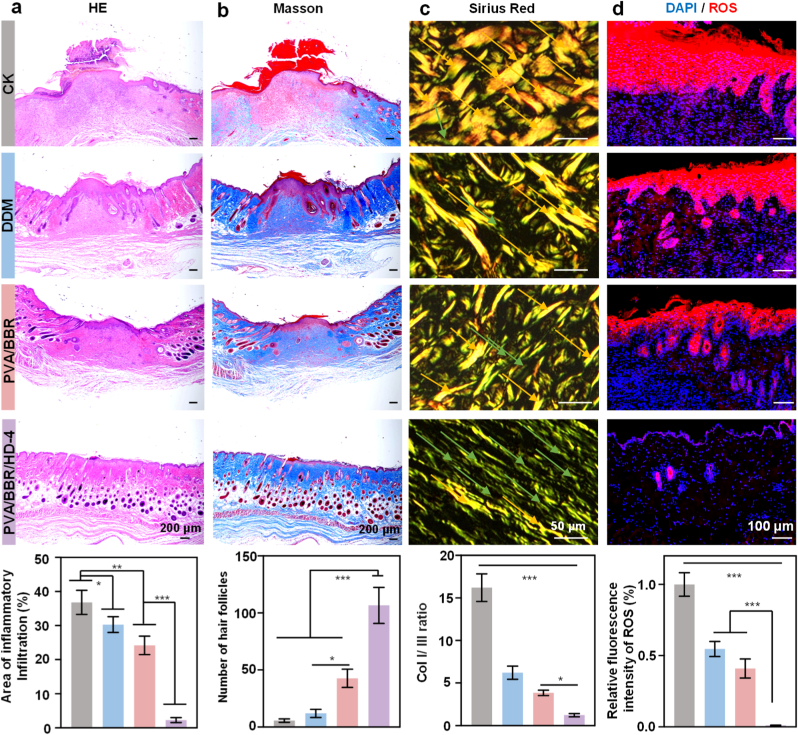
Histological and immunofluorescence analyses of diabetic wound tissues treated with different hydrogels. Representative images of (a) HE, (b) Masson's trichrome, (c) Sirius Red staining, and (d) ROS immunofluorescence staining, along with corresponding quantitative analyses on day 14 (n = 3). Statistical significance was determined using one-way ANOVA (∗*p*< 0.05, ∗∗*p*< 0.01, ∗∗∗*p* < 0.001). Error bars represent standard deviation. (For interpretation of the references to color in this figure legend, the reader is referred to the Web version of this article.)

During the late healing phase (day 14), histological differences among the groups became increasingly pronounced. Wounds treated with PVA/BBR/HD-4 exhibited more complete and well-organized skin architecture, characterized by a thinner dermis, a smooth and continuous epidermis, and markedly reduced inflammatory cell infiltration (Fig. 7a). Masson's trichrome staining revealed more uniform collagen deposition and a greater number of regenerated skin appendages in this group (Fig. 7b). Sirius red staining further confirmed a significantly reduced Col I/Col III ratio (Fig. 7c), indicating effective regulation of abnormal extracellular matrix remodeling in DIW. Given that chronic hyperglycemia in diabetes promotes sustained The reactive oxygen species (ROS) accumulation, thereby exacerbating oxidative stress and delaying wound healing, local ROS levels were also assessed [68]. The results demonstrated a significant reduction in ROS levels within wound tissues following treatment with the PVA/BBR/HD-4 hydrogel (Fig. 7d), further confirming its potent antioxidant and tissue-protective effects in the diabetic pathological microenvironment.

### 2.8 Transcriptome sequencing of diabetic infected wound repair

**Fig. 8:**
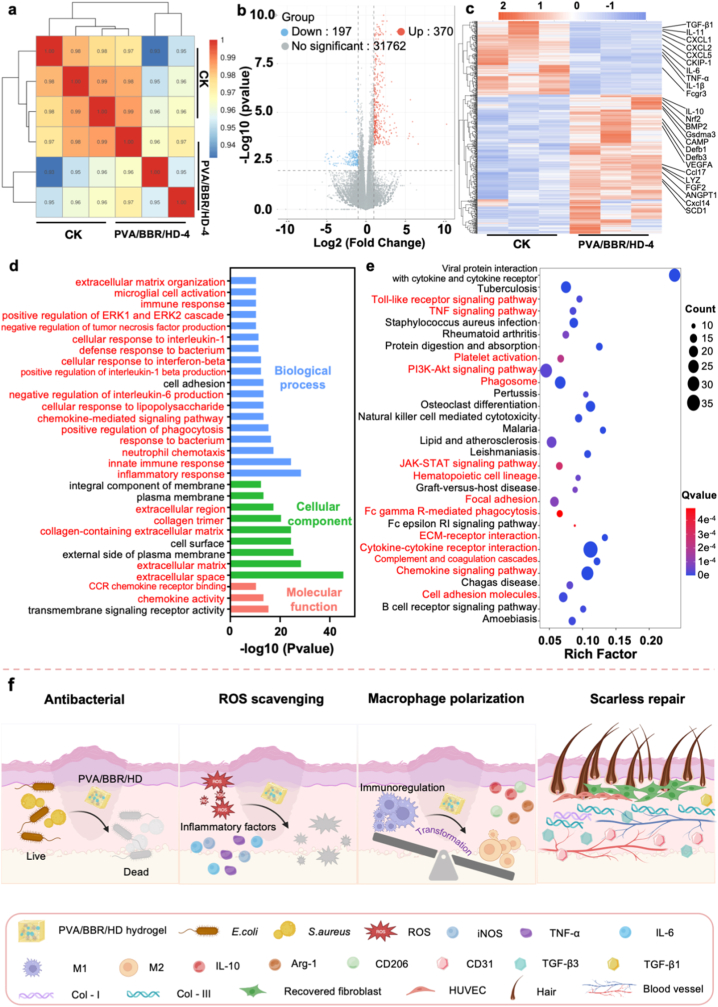
Transcriptomic analysis of diabetic wound tissues (n = 3). (a) Correlation analysis of transcriptomic datasets. (b) Volcano plot and (c) heatmap of differentially expressed genes. (d) Gene Ontology (GO) enrichment analysis and (e) KEGG pathway analysis. (f) Proposed molecular mechanism by which the PVA/BBR/HD-4 hydrogel promotes scarless wound healing.

To systematically elucidate the molecular mechanisms underlying the therapeutic effects of the PVA/BBR/HD-4 hydrogel in diabetic infected wound (DIW) healing, whole-transcriptome RNA sequencing was performed on wound tissues collected 14 days post-treatment. The PBS-treated group served as the control to identify gene expression changes specifically induced by the PVA/BBR/HD-4 hydrogel intervention. Pearson correlation analysis demonstrated strong consistency among biological replicates, with correlation coefficients (R^2^) exceeding 0.93 across all samples, confirming the high reproducibility and reliability of the sequencing data (Fig. 8a). Volcano plot analysis revealed substantial transcriptional reprogramming in response to PVA/BBR/HD-4 treatment. Compared to the control group, a total of 370 genes were significantly upregulated, while 197 genes were significantly downregulated (Fig. 8b). Functional annotation of these differentially expressed genes (DEGs) indicated pronounced activation of multiple biological processes closely associated with effective wound repair. Notably, genes involved in antimicrobial defense—including CAMP, Defb1, Defb3, and LYZ—were significantly upregulated, consistent with enhanced bacterial clearance. Angiogenesis-related genes such as VEGFA, FGF2, and ANGPT1 were also markedly increased, suggesting improved vascular regeneration. Additionally, genes associated with immune regulation and anti-inflammatory responses, including IL-10, Nrf2, Ccl17, and Cxcl14, exhibited elevated expression levels. Importantly, genes related to hair follicle development and skin appendage regeneration, such as BMP2, Gsdma3, and SCD1, were significantly upregulated, indicating activation of regenerative pathways beyond simple wound closure. In contrast, genes closely associated with fibrosis and scar formation—including TGF-β1, IL-11, and CKIP-1—were significantly downregulated following PVA/BBR/HD-4 treatment. Additionally, multiple pro-inflammatory cytokines and chemokines, such as CXCL1, CXCL2, CXCL5, IL-6, TNF-α, IL-1β, and Fcgr3, showed markedly reduced expression levels (Fig. 8c), indicating effective suppression of chronic inflammation.

Gene Ontology (GO) enrichment analysis further revealed that these DEGs were predominantly associated with biological processes related to antimicrobial defense, angiogenesis, immune regulation, and inflammatory response (Fig. 8d). These processes play critical roles in bacterial clearance, vascular remodeling, and the resolution of sustained inflammation, all of which are essential for successful DIW healing. KEGG pathway enrichment analysis revealed that treatment with the PVA/BBR/HD-4 hydrogel significantly modulated multiple signaling pathways involved in inflammation regulation, immune response, and tissue regeneration (Fig. 8e). These pathways included the JAK–STAT signaling pathway, PI3K–Akt signaling pathway, TNF signaling pathway, cytokine–cytokine receptor interaction, platelet activation, ECM–receptor interaction, Toll-like receptor signaling pathway, and chemokine signaling pathway. Collectively, these pathways play crucial roles in regulating macrophage polarization, epithelial cell migration and proliferation, angiogenesis, and extracellular matrix remodeling. In summary, the PVA/BBR/HD-4 hydrogel promotes high-quality regenerative repair of diabetic infected wounds through multi-targeted, multi-level mechanisms, thereby promoting scar-free healing outcomes in diabetic wounds (Fig. 8f).

We validated the key activation nodes of the PI3K–Akt and JAK–STAT signaling pathways at the tissue level using immunohistochemical staining for p-Akt and p-STAT3 (Fig. S15a_1-2_, b_1-2_). The results demonstrated a significant reduction in the expression of p-Akt and p-STAT3 in the treatment group, indicating effective inhibition of these inflammation-related signaling pathways. These findings suggest that the PVA/BBR/HD-4 hydrogel can modulate macrophage reprogramming from the pro-inflammatory M1 phenotype to the anti-inflammatory, reparative M2 phenotype by suppressing the PI3K–Akt and JAK–STAT pathways, thereby fostering a favorable environment for tissue repair [69,70].

### 2.9 Promoting scar-free healing in rabbit ears

We employed a rabbit ear scar model to evaluate the anti-scarring effects of the treatment (Fig. S16a). This model is characterized by minimal wound contraction and consistent formation of hypertrophic scars (HS) due to delayed re-epithelialization. It is widely regarded as one of the most classic and reliable animal models for simulating human HS. Macroscopic observations and quantitative analysis of wound closure rates demonstrated that the PVA/BBR/HD-4 hydrogel significantly accelerated wound healing, with the wound nearly completely closed by day 14 (Fig. S16b–c). In contrast, the PBS-treated group developed elevated, hard-textured pathological tissue. Notably, while distinct scarring remained visible at the center of the wound in the Beifuji and PVA/BBR groups, the scar in the PVA/BBR/HD-4 group was noticeably flattened, and its color closely resembled that of normal skin. Furthermore, the local wound pH gradually decreased from a slightly alkaline level during the initial infection phase and returned to the normal range by day 14 (Fig. S16d). These results indicate that the PVA/BBR/HD-4 hydrogel not only significantly promotes wound closure but also effectively reduces scar formation.

## 3 Conclusion

This study developed a multifunctional PVA/BBR/HD hydrogel through physical self-assembly, enabling the stable co-loading of BBR and HD. The hydrogel exhibits stable mechanical properties and excellent deformation recovery, along with tunable swelling and degradation behaviors. These characteristics allow it to maintain structural integrity in complex wound environments while meeting the requirements for sustained tissue repair. Both *in vitro* and *in vivo* experiments demonstrate that the hydrogel significantly accelerates wound closure in rabbit ear scar models, mixed bacterial infections, burn wounds, and diabetic chronic wounds. It effectively reduces bacterial load and inflammatory responses, promotes angiogenesis and tissue regeneration, and inhibits abnormal collagen deposition and scar formation. Additionally, the hydrogel's intrinsic AIE response enables visual monitoring of dynamic wound pH changes, providing an intuitive method for assessing wound status. Overall, the PVA/BBR/HD hydrogel represents a promising integrated diagnostic and therapeutic strategy for high-quality repair and intelligent management of multiple types of difficult-to-heal wounds.

## 4 Materials and methods

### 4.1 Animal ethics statement

Male C57BL/6 mice, aged 7–8 weeks and weighing 20–24 g, were used as experimental animals. All animal experiments were conducted in accordance with the ARRIVE guidelines and were approved by the Institutional Animal Care and Use Committee of Nanjing University of Science and Technology (IACUC-NJUST-2024-0718).

### 4.2 Statistical analysis

All experiments were performed in triplicate or more, with results expressed as mean ± standard deviation (SD). Statistical analyses were conducted using GraphPad Prism 9 and ImageJ software. Student's t-test was used for comparisons between two groups, while one-way ANOVA followed by Tukey's post hoc test was applied for multiple-group comparisons. Statistical significance was defined as *p*< 0.05 (∗), *p*< 0.01 (∗∗), and *p*< 0.001 (∗∗∗), while ns indicates no significant difference.

## CRediT authorship contribution statement

**Rui Fang:** Data curation, Investigation, Methodology, Visualization, Writing – original draft. **Simeng Chen:** Formal analysis, Investigation, Methodology. **Qiaozhen Liu:** Investigation, Methodology. **Mingxiong Zhang:** Data curation, Investigation. **Xi Xu:** Data curation, Investigation. **Jianfa Zhang:** Conceptualization, Funding acquisition, Project administration, Supervision, Writing – review & editing.

## Declaration of competing interest

The authors declare that they have no known competing financial interests or personal relationships that could have appeared to influence the work reported in this paper.

## Data Availability

Data will be made available on request.
