# Supplementary material for: An AIE-active polyvinyl alcohol/berberine/hemoadhican smart hydrogel for scarless wound healing with visual pH monitoring
Source: Mater Today Bio. 2026 May 10;38:103224. doi: 10.1016/j.mtbio.2026.103224 (PMC13194557; doi:10.1016/j.mtbio.2026.103224)
Supplement: Multimedia component 1 [file mmc1.docx]

Supporting Information

**An AIE-Active Polyvinyl Alcohol/Berberine/Hemoadhican Smart Hydrogel for Scarless Wound Healing with Visual pH Monitoring**

Rui Fang^a, b^, Simeng Chen^a, b^, Qiaozhen Liu^a, b^, Mingxiong Zhang^a, b^, Xi Xu^a, b^, Jianfa Zhang^a, b^*

^a^ Center for Molecular Metabolism, Nanjing University of Science & Technology

^b^ Key Laboratory of Metabolic Engineering and Biosynthesis Technology, Ministry of Industry and Information Technology

***** Address correspondence to:

Jianfa Zhang

Center for Molecular Metabolism, Nanjing University of Science & Technology, 200 Xiaolingwei Street, Nanjing, 210094, China

E-mail: [jfzhang@mail.njust.edu.cn](mailto:jfzhang@mail.njust.edu.cn)

**Supplementary Experiment**

1.1 Materials

The polysaccharide HD (molecular weight = 23,700 kDa) was extracted and purified from fermentation broth following the procedures described in our previous publication [1]. Polyvinyl alcohol (PVA 1799, 98–99%) and BBR (98%) were obtained from Macklin Biochemical Technology Co., Ltd., Shanghai, China. The reactive oxygen species (ROS) assay kit was purchased from Jiancheng Bioengineering Institute, Nanjing, China, and the CCK-8 assay kit was obtained from MedChemExpress LLC, Shanghai, China. Bacterial strains, including Staphylococcus aureus (ATCC 25923) and Escherichia coli (ATCC 25922), as well as cell lines including NIH-3T3 mouse embryonic fibroblasts, L929 mouse fibroblasts, RAW 264.7 mouse macrophages, and human umbilical vein endothelial cells (HUVECs), were obtained from the China Center for Type Culture Collection. Dulbecco’s modified Eagle medium (DMEM), fetal bovine serum (FBS), and penicillin-streptomycin (P/S) were purchased from Gibco, Grand Island, NY, USA. Levofloxacin hydrochloride (Levo, 98%) was obtained from Aladdin Chemical Co., Ltd., Shanghai, China. Urgotul silver sulfadiazine (SSD) dressings, DuoDerm™ hydrogels (DDM) and Beifuji gel were acquired from commercial sources. All reagents were of analytical grade and used as received, and all water employed in the experiments was deionized. Male C57BL/6 mice, aged 7–8 weeks and weighing 20–24 g, and adult female New Zealand White rabbits, aged 3–4 months and weighing 2–2.5 kg, were used as experimental animals. All animal experiments were conducted in accordance with the ARRIVE guidelines and were approved by the Institutional Animal Care and Use Committee of Nanjing University of Science and Technology (IACUC-NJUST-2024-0718).

1.2 Preparation of PVA/BBR/HD Hydrogels

Hydrogels were prepared following the method of Gu et al. with slight modifications [2]. Briefly, BBR was dissolved in PBS to prepare a 5% (w/v) hot solution (60 °C, pH 7.0). Subsequently, 10 mL of the BBR solution was mixed with 2 g of PVA and varying amounts of HD (0, 2, 4, or 6 g), and deionized water was added to a final volume of 100 mL. The resulting mixture was sonicated at room temperature for 10 min, then heated at 90 °C for 3 h to ensure complete dissolution and homogeneity of all components. After degassing, the solution was cast into pre-prepared molds and subjected to three consecutive freeze-thaw cycles, each consisting of freezing at −20 °C for 16 h followed by thawing at room temperature for 8 h. The resulting hydrogels were designated as PVA/BBR, PVA/BBR/HD-2, PVA/BBR/HD-4, and PVA/BBR/HD-6, respectively.

1.3 Physicochemical Characterization of PVA/BBR/HD Hydrogels

The chemical bond structures of PVA, BBR, HD, and freeze-dried hydrogel samples were analyzed using Fourier Transform Infrared Spectroscopy (FTIR; Nicolet iS20, Thermo Fisher Scientific, USA) over the range of 525–4000 cm⁻¹. Elemental composition and chemical states were characterized by X-ray photoelectron spectroscopy (XPS; ESCALAB Xi⁺, Thermo Fisher Scientific, USA). Mechanical properties were evaluated through compression testing at room temperature using an electronic universal testing machine (ETN503A, WANCE, China). Swelling behavior was determined gravimetrically [3]. *In vitro* degradation was assessed following the method described by Lu et al. [4].

1.4 Rheological Testing

The rheological properties of all hydrogel samples were measured using a rotational rheometer (Physica MCR 101, Anton Paar, Austria) equipped with parallel plate geometry (PP-50, 1 mm gap). Dynamic frequency scans were conducted at 37 °C under a constant strain of 0.1% over a frequency range of 0.1–100 Hz to determine the storage modulus (G′) and loss modulus (G″). Strain-sweep experiments were performed at 37 °C by alternating between low strain (0.1%) and high strain (100%) for 60 s each to evaluate structural stability and self-recovery behavior. Temperature ramp tests were conducted from 25 to 45 °C at a rate of 2 °C/min. Additionally, shear rate sweeps were performed at 37 °C under a constant strain of 0.1%, with shear rates ranging from 0.1 to 1000 s⁻¹, to characterize the viscosity variations of the hydrogels.

1.5 Characterization of Hydrogel Injectability

Following the method described by Lu et al., the injectability of the PVA/BBR/HD hydrogel was evaluated [4]. The hydrogel was loaded into a 1 mL syringe, and using a universal testing machine in compression mode, the force required to extrude the hydrogel through a 27 g needle was measured at a speed of 10 mm/s. The maximum force recorded during the test was defined as the injectability force.

1.6 Assessment of Injectability and Adaptive Behavior

A 200 μL sample of PVA/BBR/HD hydrogel was loaded into a 1 mL syringe to evaluate injectability by manual injection at room temperature and at 37 °C. The injection process and morphological changes of the hydrogel were documented photographically at designated time points.

To assess shape adaptability, the hydrogel was placed into a pre-fabricated irregular silicone mold, and its gradual coverage of the mold was continuously recorded over five minutes, providing a visual evaluation of its ability to conform to complex geometries.

1.7 Morphological Characterization

Freeze-dried hydrogel samples were examined using scanning electron microscopy (SEM; GeminiSEM 300, ZEISS). The samples were sputter-coated with gold for 45 s at 10 mA using a Quorum SC7620 sputter coater. SEM images were acquired at an accelerating voltage of 3 kV, and pore sizes were quantified using Fiji (ImageJ) software.

1.8 Spectral Characterization

Color images of PVA/BBR/HD hydrogels under visible light and ultraviolet (365 nm) illumination at various pH levels were captured using a smartphone, and the response times were recorded. These images were processed with color extraction software to convert them into RGB values for quantitative analysis. UV–visible absorption spectra were recorded using a UV–visible spectrophotometer (Biomate 3S, Thermo Fisher Scientific), and photoluminescence (PL) spectra were obtained with a fluorescence spectrometer (LS-55, PerkinElmer) with an excitation wavelength of 345 nm and emission scanning from 450 to 700 nm.

1.9 pH Monitoring Stability Testing

Intra-day Reproducibility: Samples of PVA/BBR/HD hydrogels, prepared independently, were selected for testing under indoor visible and ultraviolet light conditions. pH measurements were taken at three different times of the day: morning, afternoon, and evening. For each measurement, images of the hydrogels were captured, and RGB values were extracted. These RGB values were then converted to corresponding pH values using a pre-established calibration curve. The relative standard deviation was calculated to evaluate the system’s repeatability and stability.

Interference from Wound Exudates: To assess the potential interference of wound exudate on pH detection performance in complex biological environments, in vivo testing was performed using a mouse skin wound model. A PVA/BBR/HD hydrogel was applied to the wound surface, and images were captured at regular intervals over three consecutive days. The corresponding pH values were calculated using RGB analysis. Simultaneously, commercial pH indicator strips were used to measure the wound environment's pH in real time as a control.

1.10 *In Vitro* Antibacterial Activity

The antibacterial activity of the hydrogels was evaluated using the agar diffusion method [5]. Bacterial suspensions (S. aureus or E. coli) at 1.5 × 10⁸ CFU/mL were evenly spread onto LB agar plates. Sterilized hydrogel samples (8 mm in diameter, UV-irradiated for 30 min on each side) were placed on the agar surface and incubated at 37 °C for 24 h. Inhibition zones were photographed, and their diameters measured. Each experiment was conducted in triplicate.

1.11 Release Characteristics of BBR from PVA/BBR/HD Hydrogels

The release behavior of BBR from PVA/BBR/HD hydrogels was evaluated using a modified method based on previously published literature [6]. At 37 °C, 200 μL of the PVA/BBR/HD hydrogel was immersed in 2 mL of deionized water for the release study, and samples were collected at predetermined time intervals. For each sampling, 20 μL of the supernatant was mixed with 180 μL of dimethyl sulfoxide (DMSO). The absorbance of the resulting solution was measured at 350 nm using a microplate reader, and the cumulative release rate of BBR at each time point was calculated.

1.12 Time-killing curve

The minimum inhibitory concentration (MIC) of the PVA/BBR/HD hydrogel was determined for the Gram-positive bacterium *S. aureus* and the Gram-negative bacterium *E. coli*, respectively [7]. For the time-kill curve experiment, PVA/BBR/HD hydrogels at various concentrations (0.5× MIC, 1× MIC, 2× MIC, and 4× MIC) were incubated with bacterial suspensions (approximately 1 × 10⁸ CFU/mL). Samples were collected at predetermined time points, serially diluted, and evenly spread onto agar plates. After overnight incubation at 37 °C, colony-forming units (CFU) were counted to evaluate bacterial survival under different treatment conditions, and time-kill curves were plotted [8].

1.13 Biocompatibility Assessment

The hemocompatibility of the PVA/BBR/HD hydrogel was evaluated using a hemolysis assay, as described by Liu et al. [9]. Cytotoxicity was assessed in L929 cells using the CCK-8 assay kit, following the manufacturer’s instructions. For *in vivo* biocompatibility and degradation studies, six male C57BL/6 mice were randomly assigned to two groups (n = 3 per group): trauma control and hydrogel implantation. After anesthesia, the dorsal hair was shaved, and the area was disinfected with povidone-iodine. A longitudinal subcutaneous incision was made, and the experimental group received a subcutaneous implantation of PVA/BBR/HD hydrogel, while the control group underwent only the incision. Mice were euthanized on day 10 post-surgery. Tissue samples from the implantation site and major organs (heart, liver, spleen, lungs, and kidneys) were collected for histological analysis, and 200 μL of blood was drawn from each mouse for hematological testing [10].

1.14 Tube Formation and Scratch Wound Assays

Tube Formation Assay: HUVECs were used to evaluate the angiogenic effects of the hydrogels, following previously reported methods [11]. Briefly, HUVECs were co-cultured with hydrogel-containing medium (1 mg/mL) in 12-well plates for 24 h. The cells were then harvested and seeded at a density of 1.5 × 10⁴ cells/well into 96-well plates pre-coated with Matrigel, with untreated cells serving as controls. After incubation at 37 °C in 5% CO₂ for 6 h, tubular structures were imaged using an inverted fluorescence microscope (Ti2, Nikon), and the number of tubes was quantified using ImageJ software.

Scratch Wound Assay: NIH-3T3 cells were seeded at 5 × 10⁵ cells/well in 24-well plates and cultured for 24 h to form a confluent monolayer. A vertical scratch was created in each well using a 10 μL pipette tip. After washing with PBS, 500 μL of hydrogel-formulated medium was added to the experimental wells, while control wells received DMEM alone. Images of the scratch area were captured at 0, 12, and 24 h using an inverted fluorescence microscope, and the scratch closure area was analyzed using ImageJ software.

1.15 Evaluation of the Cellular Regulatory Effects of PVA/BBR/HD Hydrogel

*In vitro* antioxidant activity: The hydrogel’s antioxidant capacity was evaluated using a ROS detection kit, following the manufacturer’s instructions. RAW 264.7 cells were seeded at a density of 2 × 10⁵ cells/well in 6-well plates. After 12 h, the cells were incubated with the DCFH-DA fluorescent probe diluted in serum-free medium for 4 h. The negative control was replaced with fresh medium, the positive control was treated with H_2_O_2_ to induce oxidative stress, and the experimental group was treated with H_2_O_2_ in combination with the hydrogel extract. After 30 min of incubation at 37 °C, the fluorescence intensity of dichlorofluorescein (DCF) was measured at an excitation wavelength of 488 nm.

*In vitro* anti-inflammatory assay: RAW 264.7 cells (2 × 10⁵ cells/well) were cultured for 12 h and then co-stimulated with lipopolysaccharide (LPS, 1 μg/mL) and interferon-γ (IFN-γ, 40 ng/mL) for 24 h in the presence of hydrogel extract. Cells were harvested, total RNA was extracted and reverse-transcribed, and mRNA expression levels of TNF-α, IL-6, and IL-10 were quantified by RT-PCR. Untreated RAW 264.7 cells served as controls. Primer sequences are listed in Table S1.

Collagen-related gene expression: L929 cells were seeded in 6-well plates and cultured until they reached approximately 80% confluence. Hydrogel-formulated medium was then added, and the cells were incubated for an additional 48 h. Total RNA was extracted and reverse-transcribed, and the expression levels of type I (Col I) and type III collagen (Col III) were analyzed by RT-PCR. Primer sequences are provided in Supplementary Table S1 [12, 13].

1.16 Application of PVA/BBR/HD Hydrogel in the Healing of Wounds Infected with Mixed Bacterial Strains

C57BL/6 mice were anesthetized, and full-thickness dorsal skin defects (~8 mm in diameter) were created following previously published protocols with minor modifications [14, 15]. A 50 μL suspension containing mixed bacteria (*S. aureus* and *E. coli*, 3.0 × 10⁸ CFU/mL) was applied to the wound surface and covered with a mesh elastic bandage for 48 h. Successful infection was confirmed by visible pus formation. Levofloxacin served as the positive control, and PBS as the negative control. Wound dressings were changed every 3 days, with regular wound photography and pH monitoring. Blood samples were collected from the tail vein for hematological analysis, and body weight changes were recorded. On day 10, mice were euthanized, and wound tissues were harvested, homogenized in sterile saline, and plated for bacterial colony enumeration. Wound tensile strength was measured using an electronic universal testing machine. Wound tissues were fixed in 4% paraformaldehyde, dehydrated, paraffin-embedded, and subjected to histological analysis.

1.17 Application of PVA/BBR/HD Hydrogel in Burn Wound Healing

A deep second-degree burn model was established in male C57BL/6 mice following previously reported protocols [16, 17]. Briefly, mice were anesthetized and shaved, and an 8 mm diameter copper rod heated in boiling water was applied to the dorsal skin for 10 s to induce deep burns. Twenty-four hours post-injury, necrotic tissue was surgically excised. Wounds were treated with PVA/BBR/HD hydrogel, PBS (negative control), or SSD (positive control). Dressings were changed every 3 days, with regular wound photography and pH monitoring. Mice were euthanized in batches on days 7 and 14. Skin tissues were collected for histological analysis, including hematoxylin–eosin (HE) staining, Masson’s trichrome staining, CD31 immunohistochemistry, Sirius red staining, and immunofluorescence. At the molecular level, mRNA expression of IL-6, IL-10, TGF-β1, and TGF-β3 was quantified by RT-PCR. Primer sequences are provided in Table S1.

1.18 Application of PVA/BBR/HD Hydrogel in Diabetic Wound Healing

Diabetic mouse models were established as previously described [18]. Eight-week-old male C57BL/6 mice were fed a high-fat, high-sugar diet for 4 weeks, followed by intraperitoneal injections of low-dose streptozotocin (STZ, 30 mg/kg) for 5 consecutive days. Successful diabetes induction was confirmed by three consecutive fasting blood glucose measurements ≥ 11 mmol/L. Mice were anesthetized, shaved, and subjected to an 8 mm full-thickness dorsal skin defect. DDM served as the positive control, and PBS as the negative control. Dressings were changed every 3 days, with regular wound photography and pH monitoring. Mice were euthanized in batches on days 7 and 14. Wound tissues were collected for histological analyses, including hematoxylin and eosin (H&E) staining, Masson’s trichrome, CD31 immunohistochemistry, Sirius red staining, and immunofluorescence. RT-PCR was used to quantify tissue mRNA levels of TNF-α, Arg-1, TGF-β1, and TGF-β3.

1.19 Transcriptome Sequencing

Total RNA was extracted using TRIzol reagent following the manufacturer’s instructions. RNA concentration and integrity were assessed using the Bioanalyzer 2100 system (Agilent Technologies, USA) with the RNA Nano 6000 kit. Sequencing libraries were prepared using the VAHTS Universal V6 RNA-seq Library Prep Kit. Raw FASTQ reads were quality-filtered with Fastp (v0.24.1) to remove adapters and low-quality sequences. Clean reads were aligned to the reference genome using HISAT2. Gene expression levels were quantified as fragments per kilobase of transcript per million mapped reads (FPKM) using HTSeq. Correlation analyses were performed in R (v3.2.0) to assess biological reproducibility. Gene Ontology (GO) and KEGG pathway enrichment analyses of differentially expressed genes (DEGs) were conducted using a hypergeometric test, and significant enrichments were visualized as bar charts and bubble plots. Immunohistochemical staining for p-Akt and p-STAT3 was conducted at the tissue level to confirm the involvement of the relevant signaling pathways.

1.20 Rabbit Ear Hypertrophic Scar Model

Adult female New Zealand White rabbits (3–4 months old, weighing 2–2.5 kg) were used to establish a hypertrophic scar model on the rabbit ear. After anesthesia, full-thickness skin defects were created on each ear using an 8-mm skin punch, and the perichondrium was removed to promote hypertrophic scar formation. The wounds were treated with PBS, Beifuji gel, and PVA/BBR/HD gel, with dressings changed every 3 days. Photographs of the wounds were taken throughout the treatment period to document the healing process and scar formation.

1.21 Statistical Analysis

All experiments were performed in triplicate or more, with results expressed as mean ± standard deviation (SD). Statistical analyses were conducted using GraphPad Prism 9 and ImageJ software. Student’s t-test was used for comparisons between two groups, while one-way ANOVA followed by Tukey’s post hoc test was applied for multiple-group comparisons. Statistical significance was defined as *p* < 0.05 (*), *p* < 0.01 (**), and *p* < 0.001 (***), while ns indicates no significant difference.

**2 Supplementary Experimental Results**


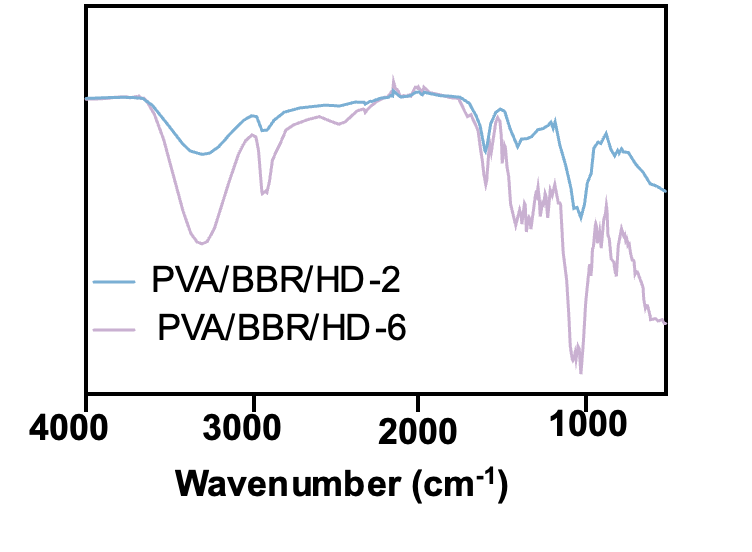


**Fig. S1** FTIR spectra of PVA/BBR/HD-2 and PVA/BBR/HD-6 hydrogel.


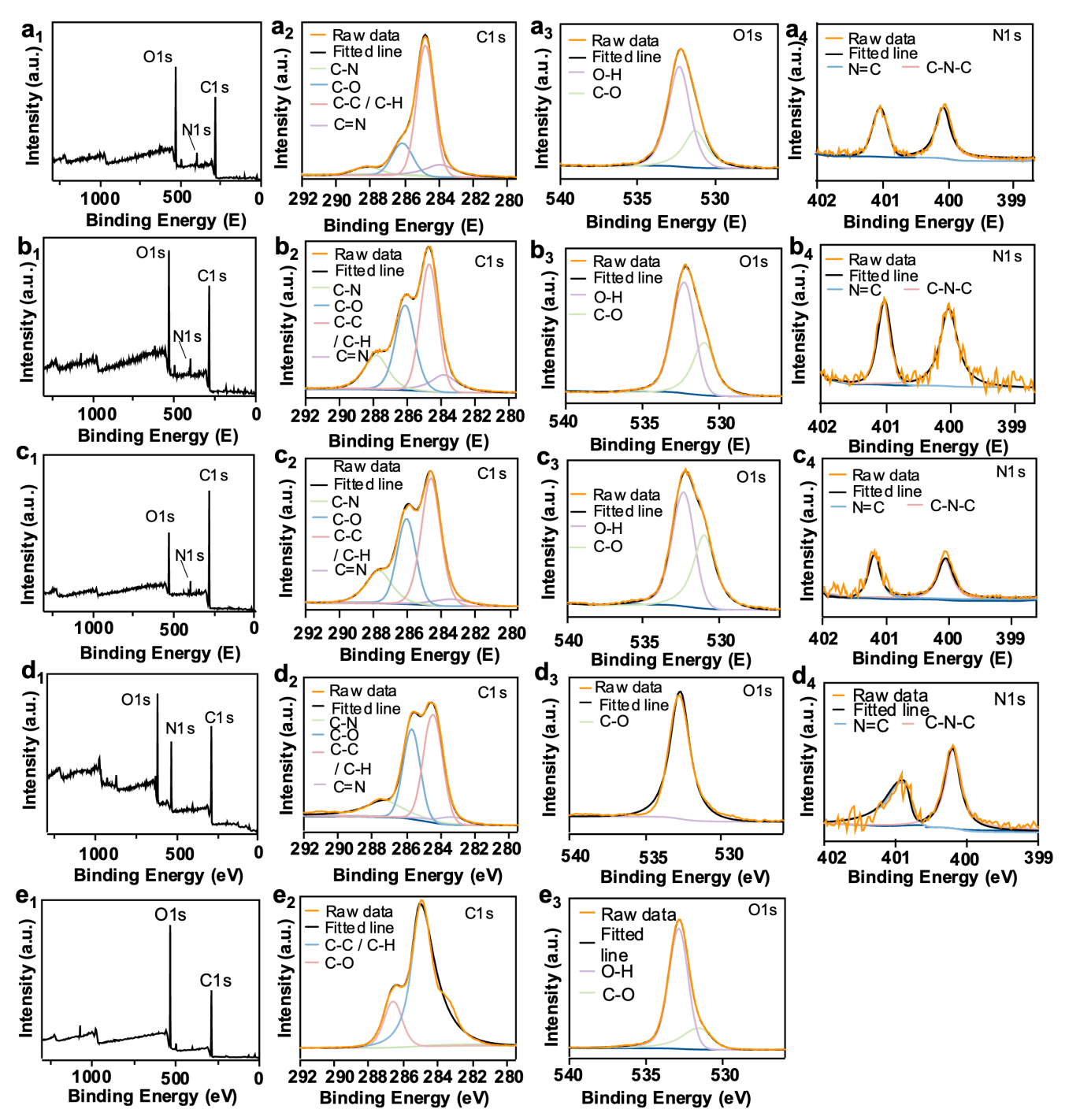


**Fig. S2** (a_1_-e_1_) XPS full survey spectra, and high-resolution XPS spectra of (a_2_-e_2_) C 1s, (a_3_-e_3_) O 1s, and (a_4_-e_4_) N 1s for the PVA/BBR (a), PVA/BBR/HD-4 (b), PVA/BBR/HD-6 (c) hydrogel, BBR (d) and HD (e).


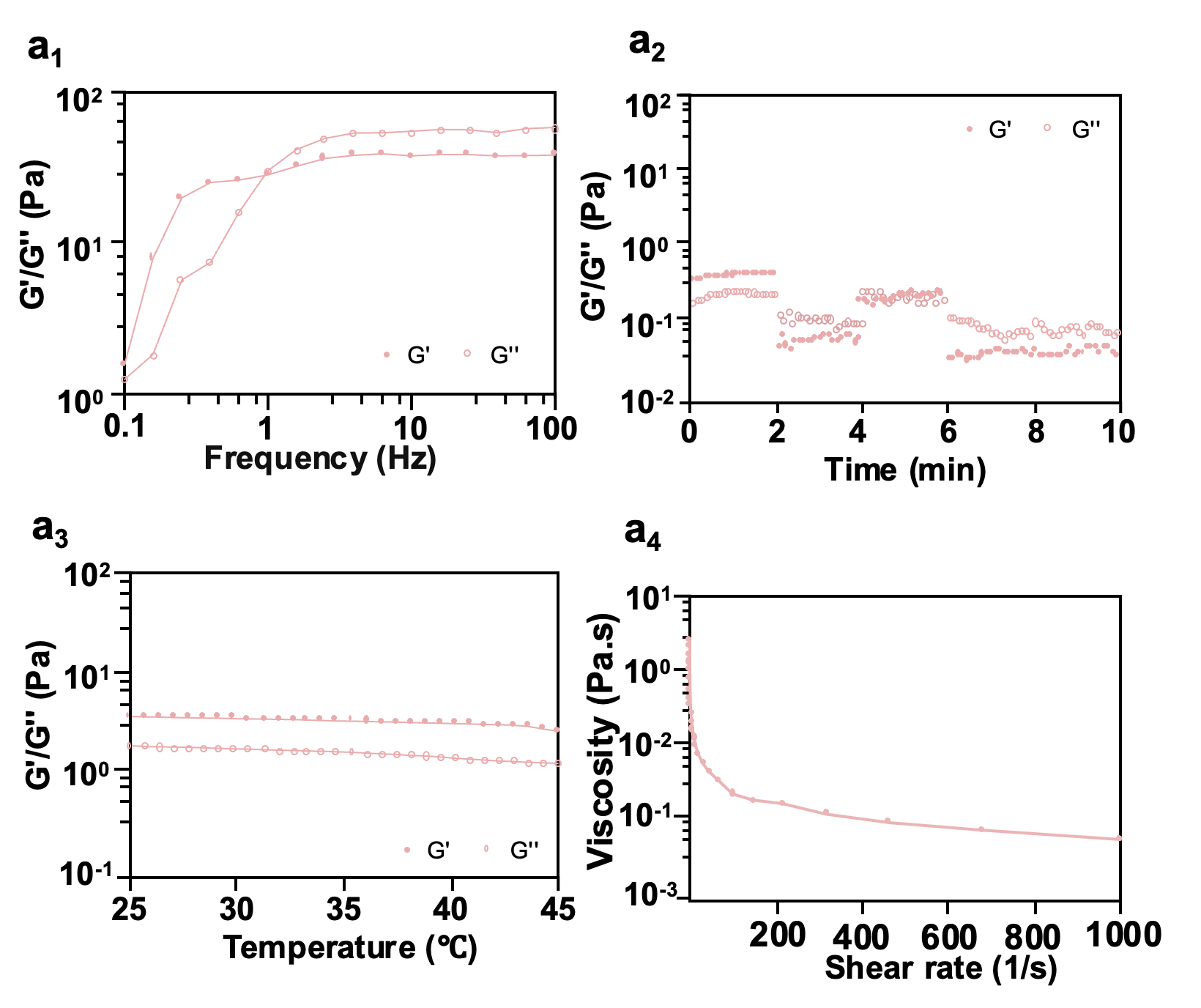


**Fig. S3** (a_1_) Storage modulus (G′) and loss modulus (G″) as functions of frequency (0.1–100 Hz). (a_2_) Cyclic strain sweep rheological test. (a_3_) Temperature-dependent storage and loss moduli (25–45 °C). (a_4_) Viscosity as a function of shear rate (0.1–1000 s⁻¹) for PVA/BBR hydrogels.


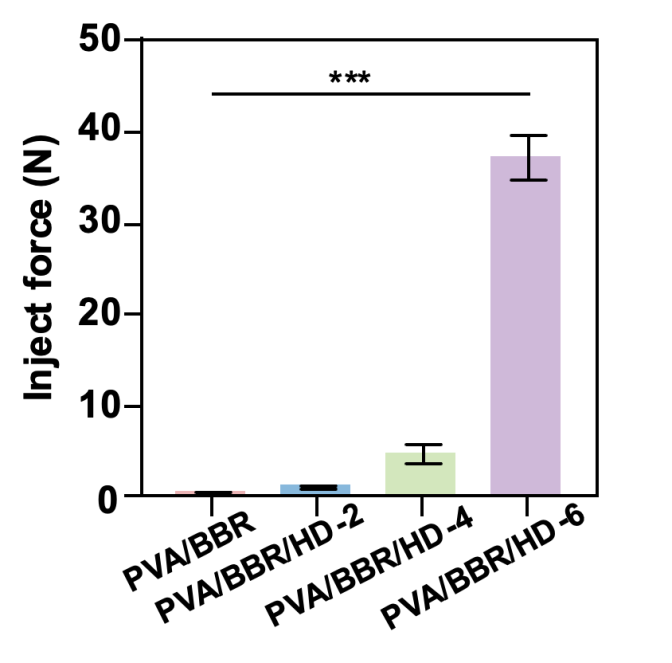


**Fig. S4** Injection force of hydrogel (n=3).


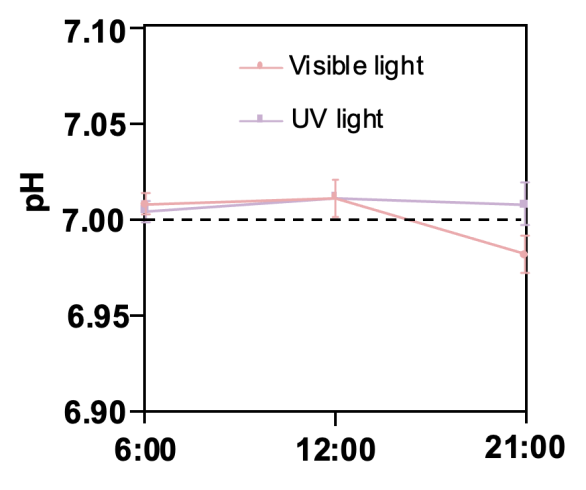


**Fig. S5** Daytime stability testing of PVA/BBR/HD-4 hydrogels for pH monitoring (n = 3).

**
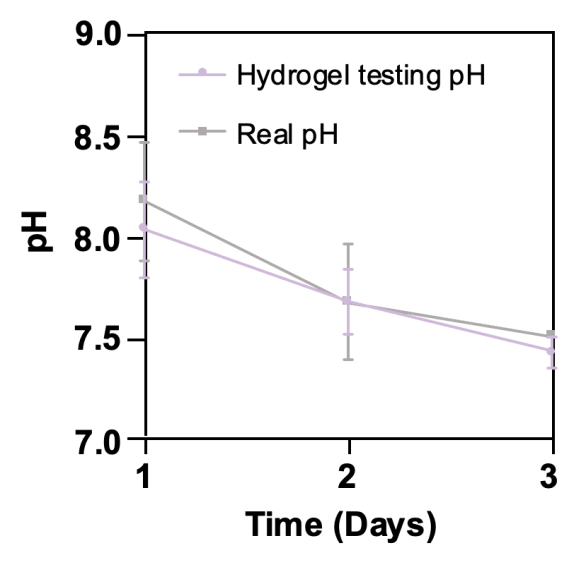
**

**Fig. S6** Comparison of actual pH values in mouse wound models with those measured in hydrogels (n = 3).

**
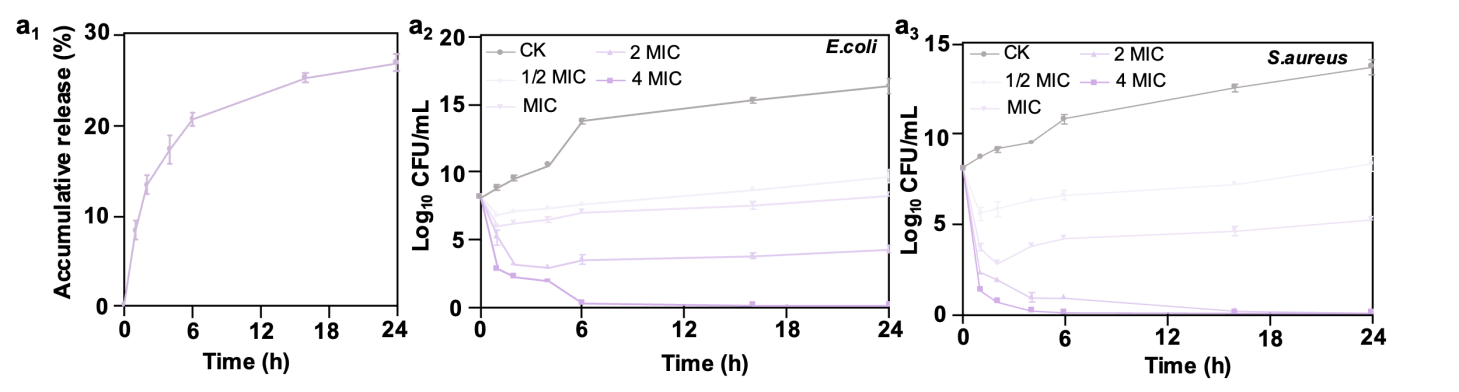
**

**Fig. S7** (a_1_) Investigation of Berberine Release Behavior. Time-kill curves of *E. coli* (a_2_) and *S. aureus* (a_3_) treated with PVA/BBR/HD-4 hydrogels at different concentrations (n=3).


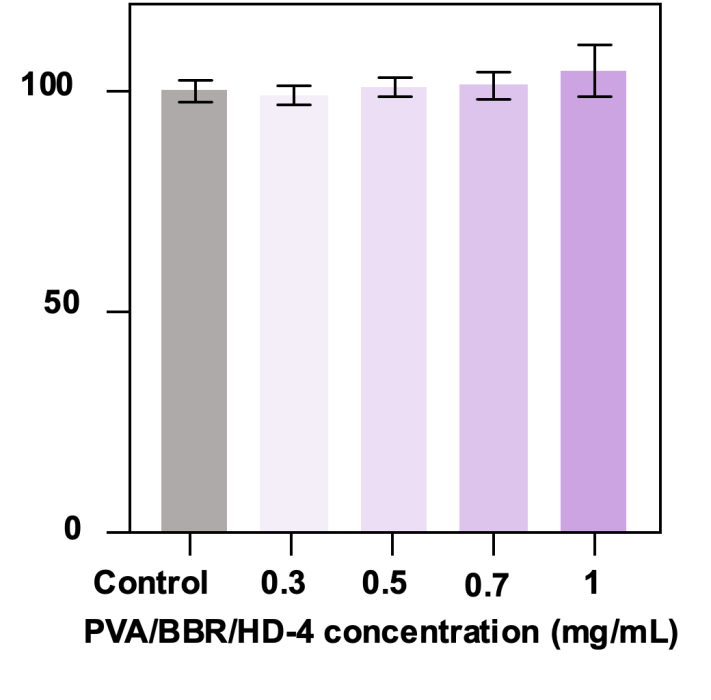


**Fig. S8** Viability of L929 cells treated with different concentrations of PVA/BBR/HD-4 hydrogel samples. (n=3)


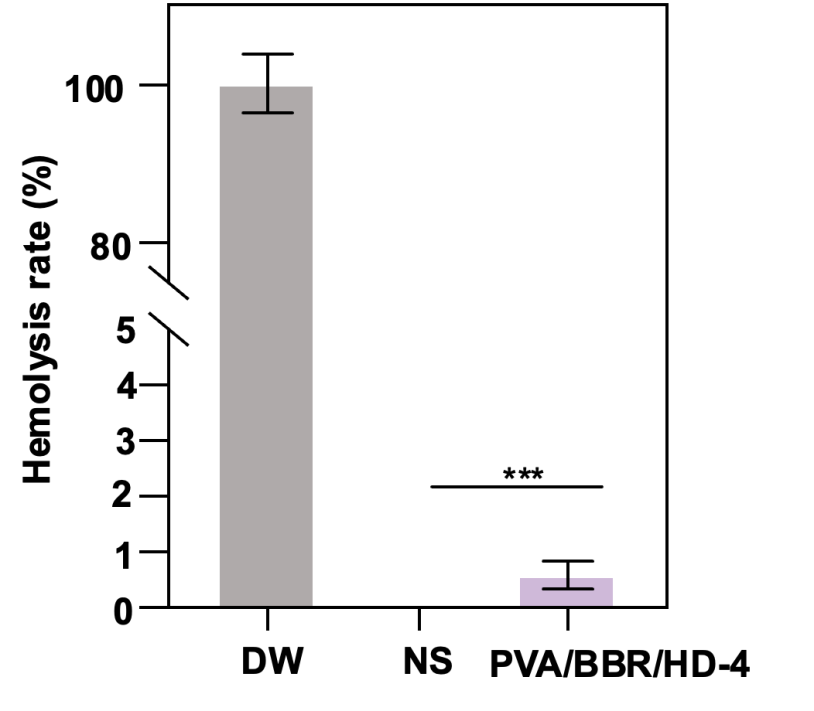


**Fig. S9** Hemolysis assay of PVA/BBR/HD-4 hydrogel treatment (n=3).


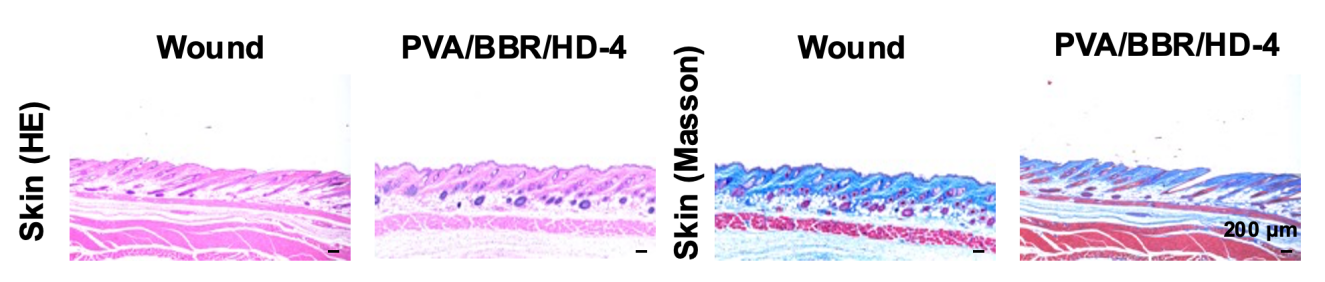


**Fig. S10** The histological changes of stained sections of skin.


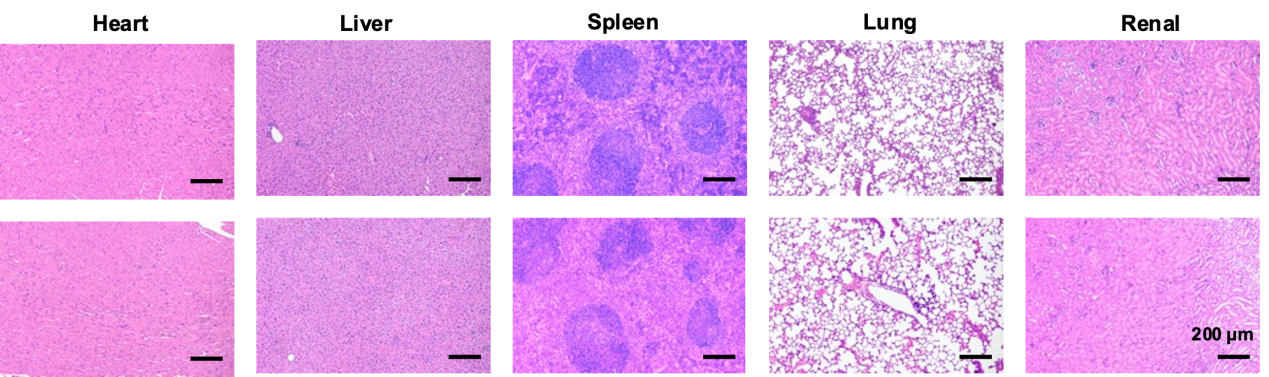


**Fig. S11** H&E staining and Masson’s trichrome staining of heart, liver, spleen, lung and kidney.


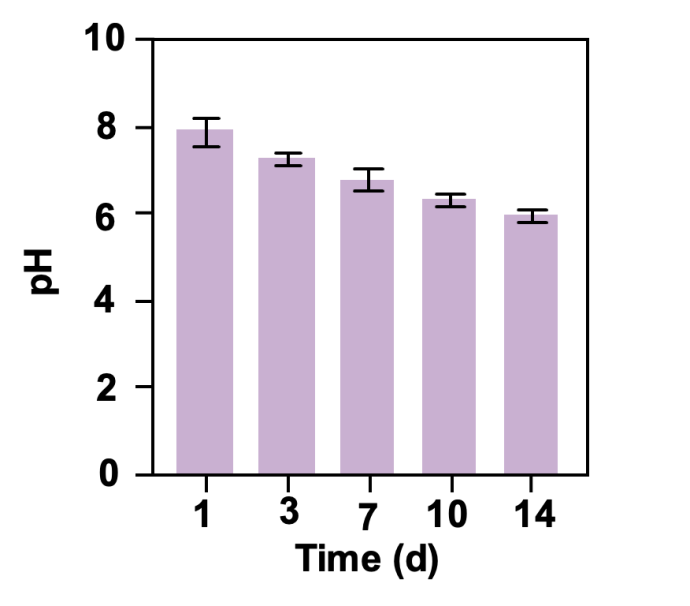


**Fig. S12** pH variation at the burn wound sites treated with PVA/BBR/HD-4 hydrogel (n = 6).


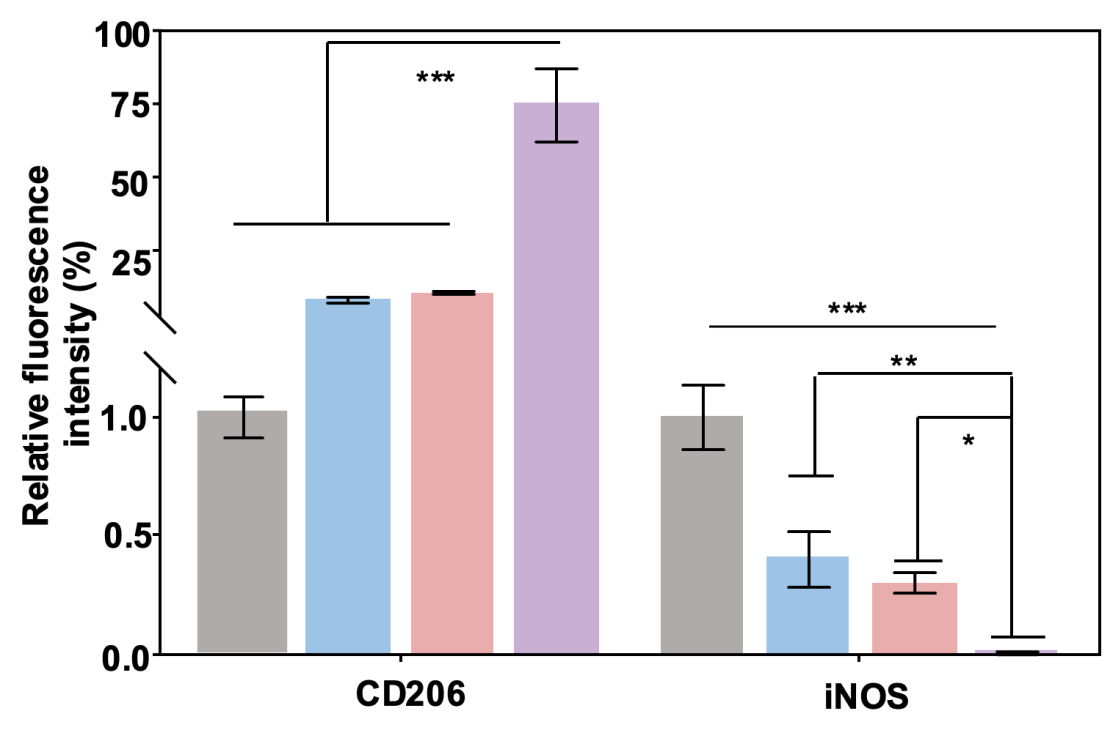


**Fig. S13** **Quantitative analysis of immunofluorescence staining** in wound tissues **on day 7** (n=3).


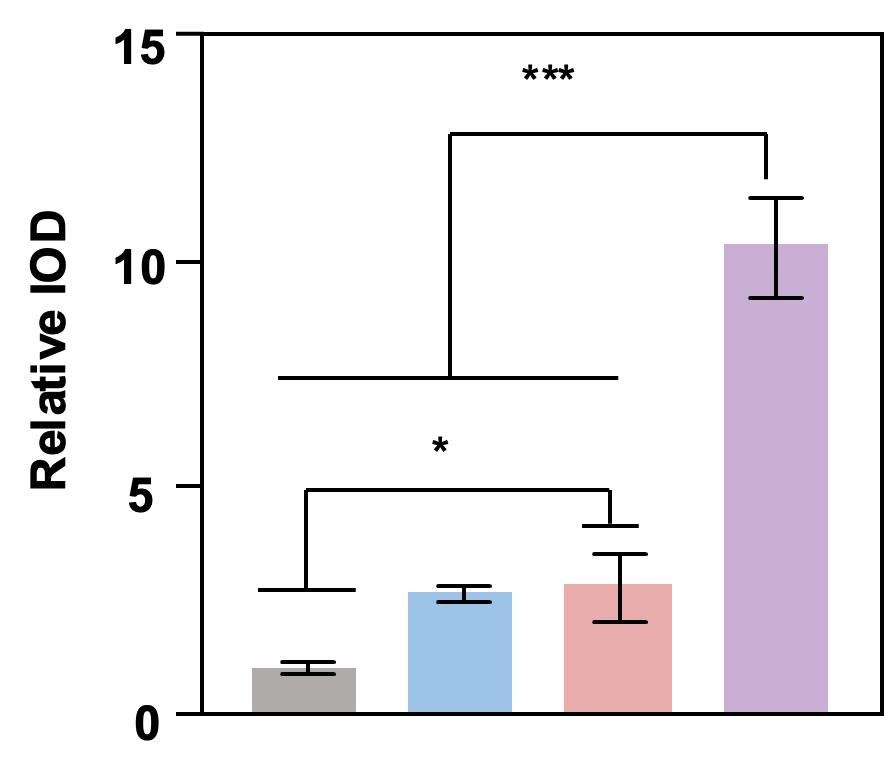


**Fig. S14** **Quantitative analysis of CD31-positive staining** in wound tissues **on day 7** (n=3).


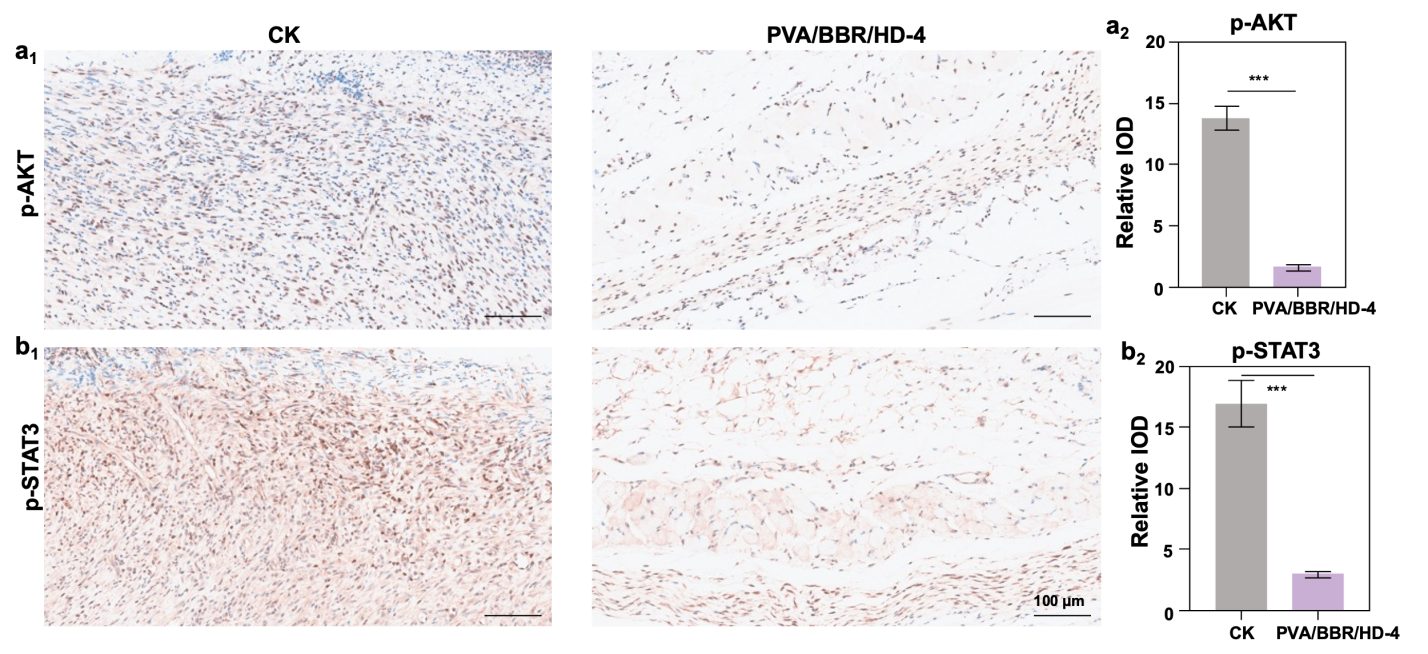


**Fig. S15** Representative images of (a_1_) p-AKT and (a_2_) corresponding quantitative analyses, as well as (b_1_) p-STAT3 and (b_2_) corresponding quantitative analyses, from immunohistochemical staining of diabetic wound tissues.


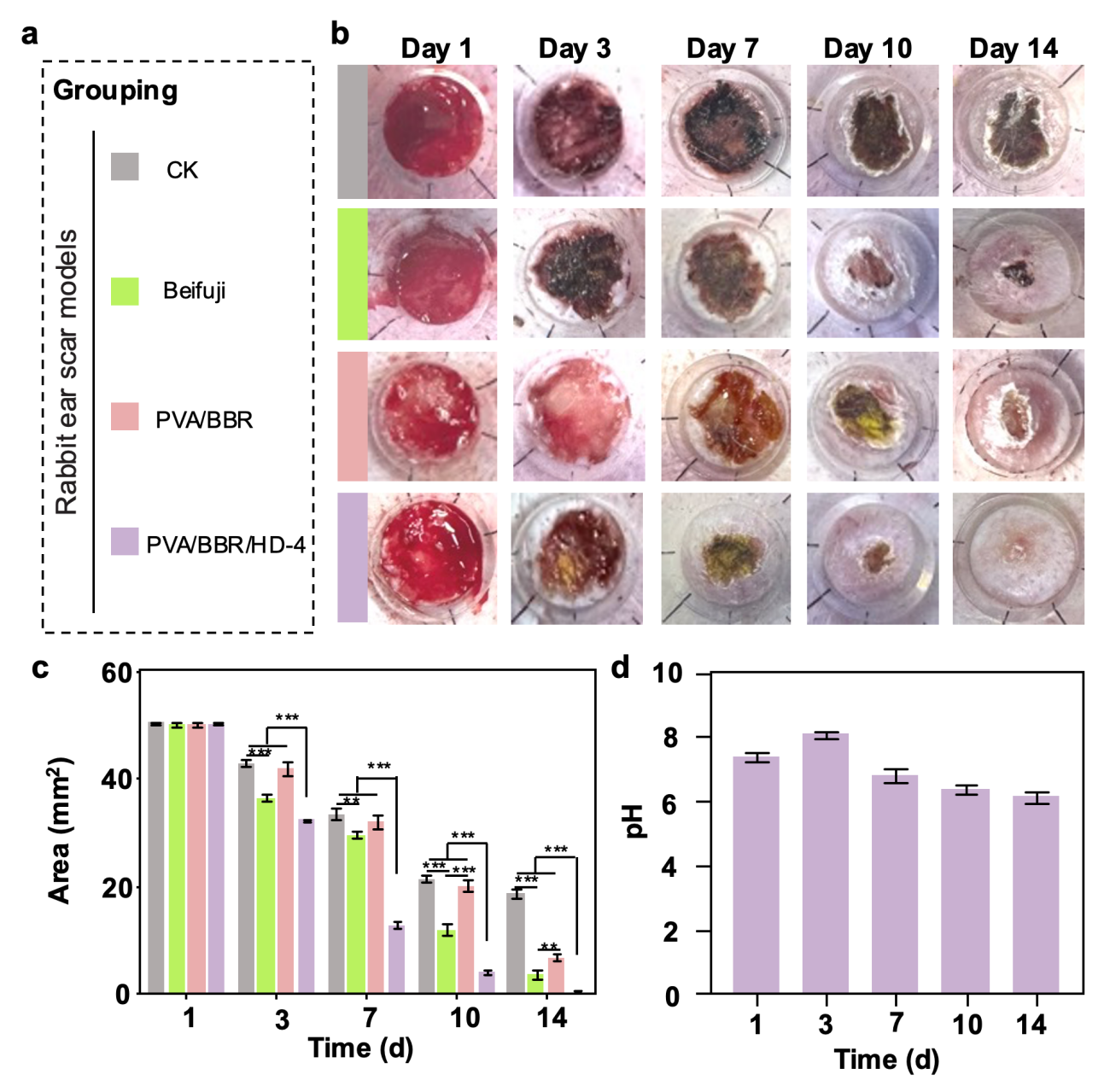


**Fig. S16** (a) Schematic diagram of rabbit ear scar model groupings. (b) Photographs of differently treated wounds on rabbit ears at various time points. (c) Quantification of wound area during healing in different groups (n = 3). (d) pH variation at infected wound sites treated with the PVA/BBR/HD-4 hydrogel (n = 3).

**Table S1.** The sequences of primers used in RT-qPCR

| **Genes** | **Forward primers (5’-3’)** | **Reverse primers (5’-3’)** |
| --- | --- | --- |
| GAPDH | CCTCGTCCCGTAGACAAAATG | TGAGGTCAATGAAGGGGTCGT |
| TNF-α | CCAGACCCTCACACTCACAA | ACAAGGTACAACCCATCGGC |
| IL-6 | CCCCAATTTCCAATGCTCTCC | CGCACTAGGTTTGCCGAGTA |
| IL-10 | AATAAGCTCCAAGACCAAGGTGT | CATCATGTATGCTTCTATGCAGTTG |
| Arg-1 | TTTCTCAAAAGGACAGCCTCG | ACAGACCGTGGGTTCTTCAC |
| Col-I | GGCAACAGCAGGTTCACCTACTC | GTCAGCACCACCAATGTCCAGAG |
| Col-III | TCTCCTGGTGCTGCTGGTCAC | TCCATGTGGTCCAACTGGTCCTC |
| TGF-β1 | CATTGCTGTCCCGTGCAGA | AGGTAACGCCAGGAATTGTTGCTA |
| TGF-β3 | CCGGATGAGCACATAGCCAA | TCTCTCTCTCCAACAGCCACTCG |

**Table S2.** Hematological and blood biochemical analyses

| **Index** | **Wound** | **J-HD** |
| --- | --- | --- |
| WBC (10^9^/L) | 13.21 ± 0.74 | 11.26 ± 0.55 |
| RBC (10^12^/L) | 8.05 ± 0.26 | 9.21 ± 0.68 |
| HGB (g/L) | 151.25 ± 19.57 | 124.99 ± 21.36 |
| HCT (%) | 42.79 ± 2.59 | 37.83 ± 4.14 |
| PLT (10^11^/L) | 339.65 ± 41.73 | 305.92 ± 52.07 |
| LYMPH (10^9^/L) | 1.58 ± 0.22 | 1.72 ± 0.31 |
| NEUT (10^9^/L) | 6.82 ± 0.93 | 6.14 ± 0.84 |
| MONO (10^9^/L) | 2.35 ± 0.29 | 2.04 ± 0.34 |

**References**

[1] C. Kong, S. Chen, X. Wang, C. Hu, B. Li, R. Fu, J. Zhang, Hemoadhican, a Tissue Adhesion Hemostatic Material Independent of Blood Coagulation, Adv. Healthc. Mater. 12(24) (2023) e2300705. <https://doi.org/10.1002/adhm.202300705>.

[2] L. Gu, X. Sun, J. Pan, D. Liu, L. Huang, Y. Yu, B. Yu, H. Cong, AIE-active PVA/berberine antibacterial hydrogel for wound healing, visual monitoring pH and dehydration, Biomaterials 323 (2025) 123432. <https://doi.org/10.1016/j.biomaterials.2025.123432>.

[3] C. Guo, L. Du, X. Du, R. Gao, X. Jiao, Z. Li, T. Zhang, P. Huang, C. Zhang, Z. Feng, ROS-responsive hydrogel loaded with berberine liposome protects against intervertebral disc degeneration by inhibiting mitochondrial fission and apoptosis, Chem. Eng. J. (2025) 167108. [https://doi.org/10.1016/j.cej.2025.167108](https://doi.org/10.1016/j.cej.2025.167108).

[4] W. Lu, X. Wang, C. Kong, S. Chen, C. Hu, J. Zhang, Hemoadhican-Based Bioabsorbable Hydrogel for Preventing Postoperative Adhesions, ACS Appl. Mater. Interfaces 16(14) (2024) 17267-17284. <https://doi.org/10.1021/acsami.4c01088>.

[5] R. Fang, N. Yu, F. Wang, X. Xu, J. Zhang, Hemoadhican Fiber Composite with Carbon Dots for Treating Severe Hemorrhage and Infected Wounds, ACS Appl. Mater. Interfaces 17(6) (2025) 9087-9102. <https://doi.org/10.1021/acsami.4c20176>.

[6] X. Liang, H. Chen, R. Zhang, Z. Xu, G. Zhang, C. Xu, Y. Li, L. Zhang, F.J. Xu, Herbal micelles-loaded ROS-responsive hydrogel with immunomodulation and microenvironment reconstruction for diabetic wound healing, Biomaterials 317 (2025) 123076. <https://doi.org/10.1016/j.biomaterials.2024.123076>.

[7] L. Huang, Y. Feng, H. Chen, L. Lai, H. Li, A. Li, J. Du, Z. Wang, T. Xin, L. Tian, S. Li, P. Zheng, C. Nie, W. Zhu, Q. Pan, Metal valence transition strategy-engineered bimetallic nanozyme thermosensitive hydrogel for deep-tissue therapy of subcutaneous abscess wounds and bacterial corneal ulcers, J. Colloid. Interface. Sci. 700(Pt 3) (2025) 138613. <https://doi.org/10.1016/j.jcis.2025.138613>.

[8] L. Wang, Q. Cai, Y. Yang, Q. Mai, Y. Zhou, Y. Liu, Y. Liu, J. Liu, Reshaping bacterial microenvironments: Hybrid biomimetic membrane-coated copper nanosystems combat bacterial biofilm infections by inhibiting bacterial quorum sensing systems, Chem. Eng. J. 512 (2025) 162088. [https://doi.org/10.1016/j.cej.2025.162088](https://doi.org/https://doi.org/10.1016/j.cej.2025.162088).

[9] Y. Liu, R. Zheng, H. Ren, J. Huang, S. Li, A self-fixing xanthan gum hydrogel membrane with ROS scavenging capability for the prevention of postoperative abdominal adhesion, Int. J. Biol. Macromol. 289 (2025) 138676. [https://doi.org/10.1016/j.ijbiomac.2024.138676](https://doi.org/https://doi.org/10.1016/j.ijbiomac.2024.138676).

[10] S. Li, X. Li, Y. Xu, C. Fan, Z.A. Li, L. Zheng, B. Luo, Z.-P. Li, B. Lin, Z.-G. Zha, H.-T. Zhang, X. Wang, Collagen fibril-like injectable hydrogels from self-assembled nanoparticles for promoting wound healing, Bioact. Mater. 32 (2024) 149-163. [https://doi.org/10.1016/j.bioactmat.2023.09.012](https://doi.org/https://doi.org/10.1016/j.bioactmat.2023.09.012).

[11] H. Xue, C. Zhang, D. Lin, Q. Gu, C. Sun, X. Lin, C. Zhang, L. Lei, L. Liu, Isoliquiritigenin micellar microneedle for pH monitoring and diabetic wound healing, Mater. Today Bio 35 (2025) 102356. [https://doi.org/10.1016/j.mtbio.2025.102356](https://doi.org/https://doi.org/10.1016/j.mtbio.2025.102356).

[12] Z. Lu, K. Tan, S. Xiang, Y. Zhang, F. Luo, X. Liu, X. Zhao, L. Ouyang, Peptide loaded self-healing hydrogel promotes diabetic skin wound healing through macrophage orchestration and inflammation inhibition, Mater. Today Bio 32 (2025) 101690. <https://doi.org/10.1016/j.mtbio.2025.101690>.

[13] P. Wang, Y. Yang, W. Zhang, J. Li, H. Shen, J. Wang, H. Zhong, S. Li, B. Chi, ERK-dependent and immunomodulatory in-situ injectable polyamino acid nanocomposite hydrogel reconstructs diabetic wound microenvironment for scarless wound healing, Chem. Eng. J. (2025) 165422. [https://doi.org/10.1016/j.cej.2025.165422](https://doi.org/10.1016/j.cej.2025.165422).

[14] S. Hou, L. Gou, F. Li, C. Li, X. Yin, P. Zhao, Y. Meng, X. Yin, Z. Xia, J. Ren, A multifunctional bacterial cellulose wound dressing based on cotton fabric for infected wound healing, Int. J. Biol. Macromol. (2025) 146850. [https://doi.org/10.1016/j.ijbiomac.2025.146850](https://doi.org/10.1016/j.ijbiomac.2025.146850).

[15] X. Yang, J. Yang, L. Wang, B. Ran, Y. Jia, L. Zhang, G. Yang, H. Shao, X. Jiang, Pharmaceutical Intermediate-Modified Gold Nanoparticles: Against Multidrug-Resistant Bacteria and Wound-Healing Application via an Electrospun Scaffold, ACS Nano 11(6) (2017) 5737-5745. <https://doi.org/10.1021/acsnano.7b01240>.

[16] L. Chai, J. Huang, M. Wang, Y. Huang, Z. Huang, R. Zhang, L. He, H. Wang, D. Chen, Y. Lei, L. Guo, Injectable deferoxamine-loaded microsphere hydrogels for inhibition of ferroptosis and promotion of third-degree burn wound healing, Mater. Today Bio 32 (2025) 101806. <https://doi.org/10.1016/j.mtbio.2025.101806>.

[17] Z. Chen, Z. Zhou, X. Zhang, Z. Wang, J. Fan, W. Wang, Y. Zheng, S. Wang, A carboxymethyl chitosan and dextran hydrogel with slow and rapid photothermal conversion for sequential promoting burn wound healing and inhibiting scar proliferation, Carbohydr. Polym. 350 (2025) 123045. <https://doi.org/10.1016/j.carbpol.2024.123045>.

[18] Z. Xiao, S. Chen, K. Zhang, S. Jiang, H. Qin, H. Jian, L. Zhou, Y. Yang, Z. Liu, W. Liu, Y. Tang, J. Luo, J. Liu, Biomimetic bilayer hydrogel loaded with SVF exosomes promotes scar-free healing of diabetic burn wounds, Chem. Eng. J. 520 (2025) 165672. [https://doi.org/10.1016/j.cej.2025.165672](https://doi.org/https://doi.org/10.1016/j.cej.2025.165672).
